# Insight into *D*_6*h*_ Symmetry: Targeting Strong Axiality in Stable Dysprosium(III) Hexagonal Bipyramidal Single‐Ion Magnets

**DOI:** 10.1002/anie.201907686

**Published:** 2019-08-22

**Authors:** Angelos B. Canaj, Sourav Dey, Emma Regincós Martí, Claire Wilson, Gopalan Rajaraman, Mark Murrie

**Affiliations:** ^1^ WestCHEM School of Chemistry University of Glasgow University Avenue Glasgow G12 8QQ UK; ^2^ Department of Chemistry Indian Institute of Technology Bombay, Powai Mumbai Maharashtra 400076 India

**Keywords:** ab initio calculations, dysprosium, hexagonal bipyramid, magnetic properties, single molecule magnets

## Abstract

Following a novel synthetic strategy where the strong uniaxial ligand field generated by the Ph_3_SiO^−^ (Ph_3_SiO^−^=anion of triphenylsilanol) and the 2,4‐di‐^t^Bu‐PhO^−^ (2,4‐di‐^t^Bu‐PhO^−^=anion of 2,4‐di‐*tert*butylphenol) ligands combined with the weak equatorial field of the ligand **L^N6^**, leads to [Dy^III^(L^N6^)(2,4‐di‐^t^Bu‐PhO)_2_](PF_6_) (**1**), [Dy^III^(L^N6^)(Ph_3_SiO)_2_](PF_6_) (**2**) and [Dy^III^(L^N6^)(Ph_3_SiO)_2_](BPh_4_) (**3**) hexagonal bipyramidal dysprosium(III) single‐molecule magnets (SMMs) with high anisotropy barriers of *U*_eff_=973 K for **1**, *U*_eff_=1080 K for **2** and *U*_eff_=1124 K for **3** under zero applied dc field. Ab initio calculations predict that the dominant magnetization reversal barrier of these complexes expands up to the 3rd Kramers doublet, thus revealing for the first time the exceptional uniaxial magnetic anisotropy that even the six equatorial donor atoms fail to negate, opening up the possibility to other higher‐order symmetry SMMs.

## Graphical abstract

**Combining a strong** linear axial ligand field with a weak equatorial ligand field, by using the polydentate ligand L^N6^, reveals for the first time the exceptional uniaxial magnetic anisotropy possible for *D*_6*h*_ symmetry. This opens up a route to further air‐stable higher‐order symmetry single‐molecule magnets.

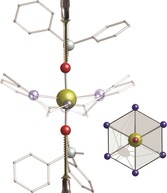

Molecular systems that display the ability to block the magnetization via an anisotropy barrier are best known as single‐molecule magnets (SMMs).^1^ The interest in these fascinating molecules is because they are among the best candidates for molecular systems that could revolutionize electron spin‐based technologies.^2^ However, the main challenge continues to be not only the fundamental ability to function at more practical temperatures (that is, above the boiling point of liquid nitrogen)^3^ but at the same time to show good air and heat stability.^4^ Recently, it has become clear that the design of high‐temperature SMMs requires strong control over the coordination environment at the level of a single metal ion.^5^ For lanthanide‐based SMMs, the magnitude of the magnetic anisotropy and the energy barrier to reorientation of the magnetization (*U*_eff_) is determined by the crystal field. Specifically, the use of the Dy^III^ ion in targeted coordination environments that promote strong uniaxial symmetry stabilizes the largest *m_J_*=±15/2 ground state and gives a large separation from the excited *m_J_* states within the energy barrier.^6^ Complexes with a symmetry belonging to an axial point group such as square antiprismatic (*D*_4*d*_),^7^ trigonal bipyramidal (*D*_3*h*_),^8^ and pentagonal bipyramidal (*D*_5*h*_),^9^ have been suggested as an effective way to favor slower relaxation of the magnetization by reducing transverse magnetic anisotropy.

However, complexes with *D*_6*h*_ symmetry remain largely unexplored. Among the very few structurally characterized mononuclear lanthanide complexes with hexagonal bipyramidal geometry only very small *U*_eff_ barriers are observed and SMM behavior is only seen on application of a dc field, owing to weak axiality and/or the presence of unwanted electron donating atoms in the equatorial plane (see Table 1).^10^

**Table 1:** Compounds with the rare hexagonal bipyramidal geometry.

Compound	Sym	*U*_eff_ [K]	*H_dc_* [Oe]	Ref.
[CeCd_3_(Hquinha)_3_(*n*‐Bu_3_PO)_2_I_3_]	*D*_6*h*_	27	1500	10b
[NdCd_3_(Hquinha)_3_(*n*‐Bu_3_PO)_2_I_3_]	*D*_6*h*_	22	2500	10b
[Yb(NO_3_)_3_(^*t*^Bu_3_PO)_2_]	*D*_6*h*_	23	1000	10c
[Dy(^*t*^Bu_3_PO)_2_(NO_3_)_3_]	*D*_6*h*_	37.1	800	10a
[Dy(^*t*^Bu_3_PO)_2_(NO_3_)_3_]	*D*_6*h*_	46.9	800	10a
**1**	*D*_6*h*_	973	0	This work
**2**	*D*_6*h*_	1080	0	This work
**3**	*D*_6*h*_	1124	0	This work

Whilst a complex with *D*_6*h*_ symmetry can theoretically provide the required strong crystal‐field splitting, the experimental realization has proven difficult, which is due to the synthetic challenges in arranging six neutral atoms in a rigid equatorial plane while at the same time engineering a strong linear axial ligand field that can offset the effect of the equatorial ligation. In this respect, we focused our efforts on generating a hexagonal bipyramidal system that would follow all the desired criteria; that is, air and heat stability, rigidity in the weak equatorial plane, and strong axial anisotropy. Using this blueprint, we report the synthesis, structure, and magnetic characterization of three novel hexagonal bipyramidal single‐ion magnets (SIMs); [Dy^III^(L^N6^)(2,4‐di‐^t^Bu‐PhO)_2_](PF_6_) (**1**), [Dy^III^(L^N6^)(Ph_3_SiO)_2_](PF_6_) (**2**) and [Dy^III^(L^N6^)(Ph_3_SiO)_2_](BPh_4_) (**3**) (Ph_3_SiO=anion of triphenylsilanol and 2,4‐di‐^t^Bu‐PhO=anion of 2,4‐di‐*tert*butylphenol), which provide a unique designed approach towards a new class of compounds with a desired hexagonal bipyramidal geometry (Figure 1, Scheme 1; Supporting Information, Figures S2, S3). Indeed, we find that [Dy^III^(L^N6^)(2,4‐di‐^t^Bu‐PhO)_2_](PF_6_) (**1**), [Dy^III^(L^N6^)(Ph_3_SiO)_2_](PF_6_) (**2**), and [Dy^III^(L^N6^)(Ph_3_SiO)_2_](BPh_4_) (**3**) are single‐ion magnets with magnetization reversal barriers that are unprecedented for the hexagonal bipyramidal geometry (see Table 1) and expand up to *U*_eff_=973 K, *U*_eff_=1080 K and *U*_eff_=1124 K for **1**, **2**, and **3**, respectively. Furthermore, we expand our study to provide detailed insight into the magnetic dynamics that governs this unique class of compounds by using ab‐initio CASSCF based computational methods.

**Figure 1:**
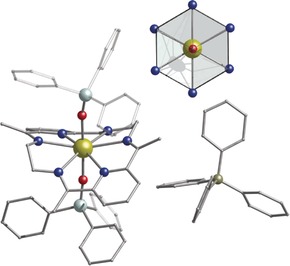
Molecular structure of **3** with Ph_3_SiO^−^ as axial ligands.^23^ Upper Inset: The highlighted hexagonal bipyramidal core. Dy gold, O red, N blue, Si light turquoise, C gray, B dark yellow. Hydrogen atoms are omitted for clarity.

**Scheme 1:**
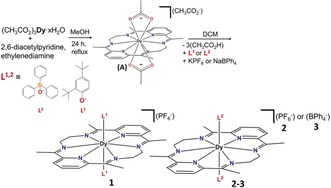
Preparation of the precursor [Dy^III^L^N6^(CH_3_CO_2_)_2_](CH_3_CO_2_)⋅9 H_2_O (**A**) and **1**–**3**.

Our strategy in synthesizing complexes **1**–**3** is shown in Scheme 1 (see the Supporting Information for details).

First, we targeted the formation of the required weak equatorial plane of the desired hexagonal bipyramid by isolating the precursor [Dy^III^L^N6^(CH_3_CO_2_)_2_](CH_3_CO_2_)⋅9 H_2_O (Supporting Information, Figure S1 and Table S1).^11^ We then sought to replace the weak axial bidentate acetate ligands with stronger anionic donors (anion of 2,4‐di‐*tert*butylphenol and anion of triphenylsilanol). Having in mind the potential importance of the second coordination sphere in controlling the magnetization reversal barrier,9f the effect of different counterions (PF_6_^−^, BPh_4_^−^) was also examined.

Compounds **1**–**3** (Figure 1; Supporting Information, Figure S2, S3) were synthesized under aerobic conditions. Refluxing 2,6‐diacetylpyridine and ethylenediamine in MeOH for 24 h in the presence of dysprosium acetate, yielded, after work up, [Dy^III^L^N6^(CH_3_CO_2_)_2_](CH_3_CO_2_)⋅9 H_2_O as single yellow crystals (Supporting Information, Figure S1 and Table S1). Substitution of the acetate groups was achieved by dissolving [Dy^III^L^N6^(CH_3_CO_2_)_2_](CH_3_CO_2_)⋅9 H_2_O in DCM, followed by the addition of the desired counter ion (PF_6_^−^, BPh_4_^−^) and the sodium salts of the preferred ligands (Ph_3_SiOH, 2,4‐di‐^t^Bu‐PhOH). For details of the experimental procedures and full characterization of **1**–**3**, see the Supporting Information.

Single‐crystal X‐ray diffraction for **1**–**3** (see the Supporting Information) reveal that in all three compounds, the dysprosium center is eight‐coordinate of a type [Dy^III^L^N6^L_2_]^+^ (L^N6^=N6‐hexagonal plane accomplished by the neutral Schiff base ligand formed from 2,6‐diacetylpyridine and ethylenediamine) with two Si−O^−^ or R−O^−^ based ligands above and below the equatorial plane, respectively (Figure 1; Supporting Information, Figures S2, S3), resulting in a strongly axial hexagonal bipyramidal geometry.

Complex **1** crystallizes in the triclinic *P* $\bar{1}$ space group (Supporting Information, Table S1), with the axial positions of the hexagonal bipyramidal geometry (ca. *D*_6*h*_ symmetry) occupied by the two bulky 2,4‐di‐^t^Bu−PhO^−^ anionic ligands (Supporting Information, Figure S2). The two strong anionic donors provide the shortest axial Dy−O bond lengths of 2.1303(14) Å and 2.1456(14) Å, while the Dy−N bonds of the hexagonal plane fall in the range of 2.5722(17) to 2.6383(17) Å (Supporting Information, Table S2) resulting in a compressed hexagonal bipyramidal geometry (Supporting Information, Figure S10).^12^ The axial O‐Dy‐O angle is 176.54(5)° while the N‐Dy‐N angles range between 80.10(5) to 103.32(6)°. The charge of the [Dy^III^(L^N6^)(2,4‐di‐^t^Bu‐PhO)_2_]^+^ cation is balanced by the presence of the PF_6_^−^ ion (Supporting Information, Figure S2). Compound [Dy^III^(L^N6^)(Ph_3_SiO)_2_](PF_6_) (**2**) crystallizes in the trigonal *R* $\bar{3}$ space group (Supporting Information, Table S1) with the axial positions of the hexagonal bipyramidal geometry employing two Ph_3_SiO^−^ anionic ligands (Supporting Information, Figure S3). The Dy−O bond lengths in **2** are 2.153(7) Å and 2.163(6) Å while the Dy−N bonds fall in the range of 2.551(6) to 2.642(6) Å (Supporting Information, Table S3). The O‐Dy‐O angle is 179.8(2)° (closer to the ideal angle of 180°) while the N‐Dy‐N angles range between 80.0(2) to 100.2(2)° resulting in a less distorted hexagonal bipyramidal geometry compared to **1** (see the Supporting Information). In contrast, the cation [Dy^III^(L^N6^)(Ph_3_SiO^−^)_2_]^+^ in **3** is stabilized by the presence of the larger BPh_4_^−^ counterion (Figure 1). Compound **3** crystallizes in the monoclinic *P*2_1_/*n* space group (Supporting Information, Table S1) with an axial O‐Dy‐O angle of 176.13(6)°. The Dy−O distances are 2.1425(16) Å and 2.1514(16) Å while the Dy−N equatorial bonds fall in the range of 2.6057(18) Å to 2.635(2) Å (Supporting Information, Table S4). The N‐Dy‐N angles range between 79.71(6)° to 97.85(6)°, stabilizing the least distorted hexagonal bipyramidal geometry in **1**–**3** (Supporting Information, Table S7, Figure S10). Additionally, analysis of the crystal packing of **1**–**3** reveals no intermolecular hydrogen bonding present, while the shortest Dy⋅⋅⋅Dy distance is 8.067 Å, 8.546 Å and 10.896 Å for **1**, **2**, and **3**, respectively (Supporting Information, Figures S11–S13).

Static (dc) and dynamic (ac) magnetic measurements via SQUID magnetometry were performed on **1**–**3**. The room‐temperature magnetic susceptibility *χ*_M_ *T* values for **1**–**3** are in close agreement with the theoretical value of 14.2 cm^3^ mol^−1^ K expected for a single non‐interacting Dy^III^ ion (^6^H_15/2_, *S*=5/2, L=5, *g*=4/3) at room temperature (Supporting Information, Figures S14–S16). Upon cooling, the *χ*_M_ *T* product decreases steadily with *χ*_M_ *T* values of 12.6 cm^3^ mol^−1^ K, 12.5 cm^3^ mol^−1^ K and 12.9 cm^3^ mol^−1^ K for **1**, **2** and **3** respectively, at 20 K, before decreasing to 10.7 cm^3^ mol^−1^ K, 11.6 cm^3^ mol^−1^ K, and 11.4 cm^3^ mol^−1^ K at 2 K (Supporting Information, Figures S14–S16). The observed decrease of *χ*_M_ *T* upon cooling is consistent with the thermal depopulation of the higher‐energy *m_J_* levels. The magnetization was also measured at 2, 4 and 6 K from 0.1–5 T for complexes **1**–**3** with the isothermal magnetization at 2 K reaching the values of 5.2, 5.3 and 5.4 μ_B_ mol^−1^, at 5 T (Supporting Information, Figures S14–S16, insets).

Variable‐temperature alternating current (ac) susceptibility measurements between 1–1488 Hz, in zero external dc field, were performed to investigate the magnetic relaxation dynamics of **1**–**3** (Figure 2 upper; Supporting Information, Figures S18–S29). The out‐of‐phase *χ*_Μ_′′ ac susceptibility data exhibit strong frequency dependent peaks with well‐defined maxima at temperatures up to 53 K, 62 K, and 74 K, in the absence of a dc field, for **1**–**3** respectively, indicating high magnetization reversal barriers. The relaxation times, *τ*, were extracted by fitting the Argand plots of *χ*_Μ_′′ vs. *χ*_Μ_′ using the generalized Debye model (Supporting Information, Figures S23, S26, S29).^13^ The *α* parameters found are in the range of 0–0.34 (2–53 K) for **1**, 0.05–0.37 (2–62 K) for **2** and 0–0.5 (2–74) for **3**. The relatively wide distribution of relaxation times in **1**–**3** is indicative of multiple relaxation processes present. Fitting the full temperature range data to the equation *τ* ^−1^=*τ*_QTM_^−1^+CT^n^+*τ*_0_^−1^exp(−*U*_eff_/*T*), in which C and n are parameters of the Raman process and *τ*_QTM_ is the rate of the quantum tunneling of magnetization (QTM),6a, 13, 14 gives energy barriers of *U*_eff_=973 K, *U*_eff_=1080 K and *U*_eff_=1124 K for **1**–**3** respectively (*n* ^(1)^=2.50, *C* ^(1)^=0.37 K^−*n*^ s^−1^, *τ*_QTM_^(1)^=0.0030 s, *τ*_0_^(1)^=0.317×10^−11^; *n* ^(2)^=2.32, *C* ^(2)^=0.34 K^−*n*^ s^−1^, *τ*_QTM_^(2)^=0.025 s, *τ*_0_^(2)^=0.196×10^−10^ s and *n* ^(3)^=2.95, *C* ^(3)^=0.014 K^−*n*^ s^−1^, *τ*_QTM_^(3)^=0.016 s, *τ*_0_^(3)^=0.152×10^−10^ s; Figure 2 lower; Supporting Information, Figures S30–S32). The values of the pre‐factor *τ*_0_, *C*, and *n* are within the observed range for Dy^III^ SMMs8b, 15 with the exponent *n* of the Raman process having a smaller value than expected for a Kramers ion (*n*=9). However, it has been reported that smaller *n* values suggest the presence of Raman processes involving optical acoustic phonons.^16^ Demagnetization through higher excited *m_J_* states is a relatively rare phenomenon that is mainly observed in low coordinate lanthanide compounds.^5^ To the best of our knowledge, these are the largest magnetization reversal barriers reported for air stable high‐coordinate dysprosium compounds illustrating the potential of the rare hexagonal bipyramidal geometry.

**Figure 2:**
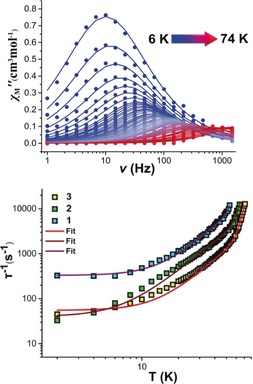
Upper: Plots of *χ*_Μ_′′(*v*) in zero applied dc field in the temperature range of 6–74 K for **3**. Lower: Temperature dependence of the relaxation time for **1** (blue squares), **2** (green squares), and **3** (yellow squares), where the solid lines are fits of the data using the parameters given in the text.

In light of the large *U*_eff_ barriers of **1**–**3**, magnetization versus field hysteresis loops were also examined. Using a slow average sweep rate of 4 mT s^−1^ (Figure 3; Supporting Information, Figures S33, S34), all of the compounds show a zero‐field step with butterfly‐like hysteresis loops which remain open at higher fields (|H|>0). At lower fields and on approaching *H*=0 the magnetization suddenly drops, indicating a strong contribution of faster relaxation effects (QTM). This is in good agreement with the very small temperature dependence between 2–10 K in the *χ*_Μ_′′(*v*) curves for **1**–**3** (Supporting Information, Figures S22, S25, S28), and the rapid increase in the *χ*_Μ_′ and *χ*_Μ_′′ versus temperature plots at lower temperatures (Supporting Information, Figures S18–S20).^17^ To provide insight into the mechanism that governs the magnetic relaxation, we have performed ab initio calculations using the CASSCF/RASSI‐SO/SINGLE_ANISO approach implemented in MOLCAS 8.2 (see the Supporting Information for details).^18^ For **1**–**3** the eight Kramers Doublets (KDs), corresponding to the ^6^H_15/2_ ground state of the Dy^III^ ion, span an energy range of about 1800 K (Supporting Information, Tables S8–S10). We find that the ground state (*m_J_*=±15/2) is highly anisotropic for **1**–**3** (g_*zz*_=19.978, 19.992 and 19.979 respectively, Table S8‐S10) with negligible transverse components (g_*xx*_=0.001, g_*yy*_=0.002 for **1**, g_*xx*_=0.001, g_*yy*_=0.002 for **2** and g_*xx*_=0.001, g_*yy*_=0.001 for **3**) establishing a strong magnetic anisotropy axis. The main magnetic anisotropy axis is nearly collinear with the pseudo *C*_6_ axis lying along the axial Dy−O bonds (Figure 4; Supporting Information, Figures S35, S36). This can be explained with the LoProp^19^ charges computed using the CASSCF wavefunction (Supporting Information, Figures S38–S40). The charge on the axial oxygen atoms is found to be nearly four times larger compared to the nitrogen atoms of the L^N6^ ligand (Supporting Information, Table S11). Importantly, this is fully consistent with our synthetic strategy of stabilizing longer Dy−N bonds in the equatorial plane and then replacing the weak axial acetate groups (Figure 1; Supporting Information, Figures S1–S3) with stronger anionic donors (anion of 2,4‐di‐*tert*butylphenol and anion of triphenylsilanol). The use of the rigid and robust L^N6^ ligand is deliberate; that is, the planar pyridine rings along with the C=N bonds in the macrocyclic ring introduces strong steric hindrance and minimizes the effect of the N donor atoms in the equatorial plane, reducing transverse anisotropy and maximizing the axial crystal field parameters in **1**–**3**. To verify this we carefully modulated the equatorial ligand environment in silico; we created three model systems **1 a**, **2 a** and **3 a** (Supporting Information, Figure S41) where the L^N6^ ligand is replaced with six less bulky NH_3_ groups. For **1 a**–**3 a** the transverse anisotropy is significantly enhanced (owing to the stronger donating NH_3_ groups) leading to a dramatic reduction of the *U*_cal_ values (606 K for **1 a**, 629 K for **2 a** and 694 K for **3 a**; Supporting Information, Figure S42, Tables S13–S15). This highlights the importance of our synthetic approach that is, minimizing the effect of the N donor atoms in the equatorial plane, which can be implemented further for the isolation of new *D*_nh_ systems.

**Figure 3:**
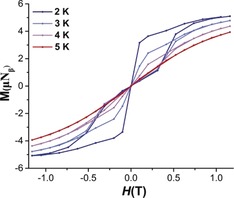
Powder magnetic hysteresis measurements for **3** with an average sweep rate of 4 mT s^−1^.

**Figure 4:**
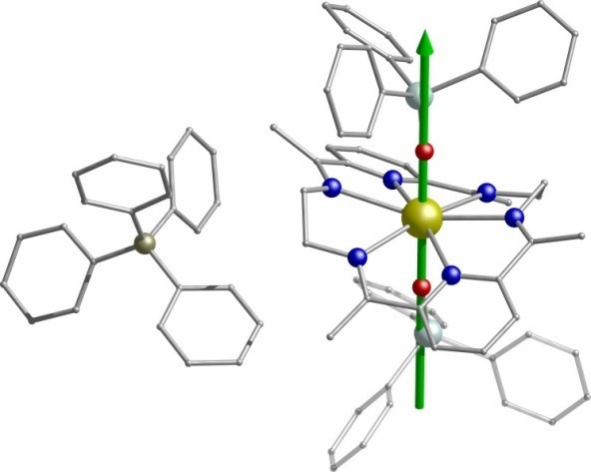
The direction of the principal anisotropy axis of the ground Kramers doublet for **3**. Dy gold, O red, N blue, Si light turquoise, C gray, B dark yellow. Hydrogen atoms are omitted for clarity.

For complexes **1**–**3** *m_J_*=±15/2 is the ground state with *m_J_*=±13/2 as the first excited state (KD2) and *m_J_*=±11/2 as the second excited state (KD3). The two excited states (*m_J_*=±13/2, and *m_J_*=±11/2; Supporting Information, Tables S8–S10) are also axial in nature and are found to lie at 620 K and 1074 K for **1**; 642 K and 1138 K for **2** and 672 K and 1208 K for **3**, above the ground state. It is important to note the relatively larger g_*xx*_/g_*yy*_ values obtained for the second excited state (*mJ*=±11/2, g_*xx*_=0.677, g_*yy*_=1.923, g_*zz*_=12.687 for **1**; g_*xx*_=0.614, g_*yy*_=1.695, g_*zz*_=12.766 for **2** and g_*xx*_=0.117, g_*yy*_=0.731, g_*zz*_=13.342 for **3**) yield larger magnetic moment matrix elements of 0.48 μ_B_, 0.42 μ_B_ and 0.17 μ_B_, which is sufficient to promote magnetic relaxation via this state, giving the maximum calculated magnetization reversal barriers of *U*_cal_=1074 K, *U*_cal_=1138 K and *U*_cal_=1208 K for **1**–**3** respectively (Figure 5; Supporting Information, Figure S37). Notably, a small transverse magnetic moment is calculated for the first two KDs (KD1, 0.72×10^−3^ μ_B_, KD2, 0.76×10^−1^ μ_B_ for **1** Figure S37 upper; KD1, 0.57×10^−3^ μ_B_, KD2, 0.64×10^−1^ μ_B_ for **2**: Supporting Information, Figure S37 lower; and KD1, 0.20×10^−3^ μ_B_, KD2, 0.49×10^−1^ μ_B_ for **3**: Figure 5) suggesting the presence of weak QTM. In **1**–**3** the calculated and experimental magnetization reversal barriers are in close agreement (1074 K and 973 K for **1**; 1138 K and 1080 K for **2** and 1208 K and 1124 K for **3**, respectively) with a small deviation probably attributed to the presence of QTM. Additionally, the crystal field parameters have been computed (Supporting Information, Table S12) to give further insight into the differences in the behavior of **1**–**3**. Using the SINGLE_ANISO code *Ĥ*_CF_=∑ ∑qk=-q Bqk O˜qk where O˜qk and Bqk are the computed extended Stevens operators and crystal field (CF) parameters, it is evident that the ratio of the non‐axial crystal field term (Bqk , where *q*≠0 and *k*=2, 4, and 6) to the axial term (Bqk , where *q*=0 and *k*=2, 4, and 6) is found to be smaller for **3**, compared to **1** and **2**, confirming the lower operational QTM and the larger relaxation barrier for **3**. Furthermore, in an attempt to evaluate how strongly the *U*_cal_ values of **1**–**3** are related to the different counter ions (PF_6_^−^, BPh_4_^−^), three new calculated model systems **1 b, 2 b**, and **3 b** were examined where the counter‐ions are not included in the ab initio calculations (Supporting Information, Table S18–S22). The *U*_cal_ values of **1 b**–**3 b** (Supporting Information, Figure S43) are very close to the *U*_cal_ values of **1**–**3** (Figure 5; Supporting Information, Figure S37) suggesting that the rigid and robust L^N6^ ligand minimizes the effect of the second coordination sphere on the magnetization reversal barrier.9f On the other hand, of particular interest is the possibility of further axial substitution. In this regard, we have developed a new calculated model (that is, **3 c**) where the two axial ligands in **3** have been replaced by the less bulky F^−^ ions, model **3 c** (Supporting Information, Figure S44). For the model system **3 c** the calculated energy barrier, *U*_cal_ is estimated at 1194 K (Supporting Information, Table S23). Importantly, model **3 c** is an extremely attractive target system since it should allow further coordination through the axial F^−^ ions, opening up a new route to 3d–4f complexes and/or magnetic chains with very high magnetic anisotropy.^20^

**Figure 5:**
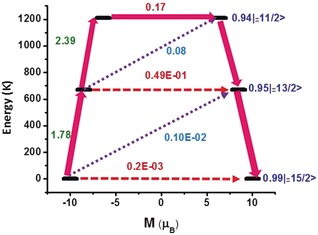
Ab initio calculated relaxation dynamics for complex **3**. The arrows show the connected energy states with the number representing the matrix element of the transverse moment (see text for details). The black line indicates the KDs as function of magnetic moments. The red dashed arrow represents QTM (QTM=quantum tunneling of the magnetization) via ground state and TA‐QTM (TA‐QTM=thermally assisted QTM) via excited states. The violet dotted arrow indicates possible Orbach process. The pink thick arrow indicates the mechanism of magnetic relaxation. The numbers above each arrow represent corresponding transverse matrix elements for the transition magnetic moments.

In summary, by using a carefully designed step‐by‐step synthetic approach we have presented a new class of compounds with the rare hexagonal bipyramidal geometry with strongly axial crystal fields. Complexes [Dy^III^(L^N6^)(2,4‐di‐^t^Bu‐PhO)_2_](PF_6_) (**1**), [Dy^III^(L^N6^)(Ph_3_SiO)_2_](PF_6_) (**2**) and [Dy^III^(L^N6^)(Ph_3_SiO)_2_](BPh_4_) (**3**) are air‐stable single‐ion magnets, exhibiting slow relaxation of magnetization through large (*ca*. 1800 K) multilevel barriers via the 3rd Kramers doublet with *U*_eff_=973 K, 1080 K and 1124 K for **1**, **2** and **3**, respectively. Our unique synthetic strategy produces air stable hexagonal bipyramidal architectures that generate pronounced axial magnetic anisotropy. Our ongoing efforts are focused on fully realizing the enormous synthetic flexibility in the design that these ≈*D*_6*h*_ complexes offer for tuning the axial and equatorial crystal fields, in order to modulate and further improve the relaxation dynamics. The linking ethylene diamine groups could be tuned to *ortho*‐phenyldiamine or 1,2‐diphenylethylenediamine that will potentially make L^N6^ even more robust and withdraw electron density from the equatorial N‐donor atoms to boost the magnetic anisotropy.^21^ Furthermore, we believe that this step‐by‐step synthetic approach can be implemented further for the isolation of new *D*_n*h*_ systems.^22^

## Conflict of interest

The authors declare no conflict of interest.

## Supporting information

As a service to our authors and readers, this journal provides supporting information supplied by the authors. Such materials are peer reviewed and may be re‐organized for online delivery, but are not copy‐edited or typeset. Technical support issues arising from supporting information (other than missing files) should be addressed to the authors.

Supplementary
