# Supplementary material for: Insight into D 6h Symmetry: Targeting Strong Axiality in Stable Dysprosium(III) Hexagonal Bipyramidal Single‐Ion Magnets
Source: Angew Chem Int Ed Engl. 2019 Aug 22;58(40):14146–51. doi: 10.1002/anie.201907686 (PMC6790654; doi:10.1002/anie.201907686)
Supplement: Supplementary file 1 — Supplementary [file ANIE-58-14146-s001.pdf]

Angewandte Chemie
Eine Zeitschrift der Gesellschaft Deutscher Chemiker

Supporting Information

Insight into D_{6h} Symmetry: Targeting Strong Axiality in Stable Dysprosium(III) Hexagonal Bipyramidal Single-Ion Magnets

Angelos B. Canaj, Sourav Dey, Emma Regincós Martí, Claire Wilson, Gopalan Rajaraman,* and Mark Murrie**

anie_201907686_sm_miscellaneous_information.pdf

Author Contributions

A.B.C. Conception: Lead; Experimental, Magnetic and Structural Investigation: Lead; Design: Lead; Acquisition of data: Lead; Project administration: Lead; Methodology: Lead; Visualization: Lead; Writing-review and editing: Equal.

E.R.M. Investigation: Supporting; Acquisition of data: Supporting; Experimental, Magnetic and Structural Investigation: Supporting.

S.D. Computational Investigation: Lead; Writing-review and editing: Equal.

C.W. Investigation: Supporting; Writing-review and editing: Equal.

G.R. Computational Supervision: Lead; Writing-review and editing: Equal.

M.M. Supervision: Lead; Funding acquisition: Lead; Conception: Lead; Writing-review and editing: Equal.

Table of Contents

1. Materials and physical measurements S4
2. Synthesis and characterization S4
3. Magnetic characterization S16
4. Ab initio calculations S34

1. Materials and physical measurements

All manipulations were performed under aerobic conditions, using materials as received. Elemental analyses (C, H, N) were performed by the University of Glasgow microanalysis service. Compound $[Dy^{III}L^{N6}(CH_3CO_2)_2](CH_3CO_2)\cdot 9H_2O$ was prepared according to a similar synthetic procedure as described in the literature.[1] Variable-temperature, solid-state direct current (dc) magnetic susceptibility data down to 2.0 K were collected on a Quantum Design MPMS-XL SQUID magnetometer equipped with a 5 T DC magnet at the University of Glasgow. Polycrystalline samples were embedded in eicosane and diamagnetic corrections were applied to the observed paramagnetic susceptibilities using Pascal's constants. Powder XRD measurements were collected on freshly prepared samples of $[Dy^{III}L^{N6}(CH_3CO_2)_2](CH_3CO_2)\cdot 9H_2O$ and **1-3** on a PANalytical X'Pert Pro MPD diffractometer (λ ($CuK\alpha_1$) = 1.5405 Å) on a mounted bracket sample stage, at the University of Glasgow. Single Crystal X-Ray diffraction data were collected using a Bruker D8 VENTURE diffractometer equipped with a Photon II CPAD detector, with an Oxford Cryosystems N-Helix device mounted on an IµS 3.0 (dual Cu and Mo) microfocus sealed tube generator at the University of Glasgow. Thermogravimetric analysis was performed on a SDT Q600 with an argon atmosphere (100 ml min^{-1}) in the 20 - 800 K temperature range (10 K min^{-1}), at the University of Glasgow.

2. Synthesis and characterization

Synthetic strategy applicable to $\mathbf{[Dy^{III}L^{N6}(CH_3CO_2)_2](CH_3CO_2)\cdot 9H_2O}$:

2,6-Diacetylpyridine (163 mg, 1 mmol), $(CH_3CO_2)_3Dy$ (171 mg, 0.5 mmol) and ethylenediamine (0.067 ml, 1 mmol) were combined in 20 ml of MeOH. After 10 min of stirring the reaction was refluxed for 24 hours giving a clear brown solution. Removing the solvent under vacuum produces thick dark brown oil. The oil was dissolved in 10 ml of CH_3Cl and by diffusion with Et_2O at ~ 5 ^{o}C $[Dy^{III}L^{N6}(CH_3CO_2)_2](CH_3CO_2)\cdot 9H_2O$ was isolated as yellow crystals. Elemental Anal. calcd (found) for $[Dy^{III}L^{N6}(CH_3CO_2)_2](CH_3CO_2)\cdot 7H_2O$: C 40.05 (40.03), H 6.05 (5.88), N 9.76 (10.0) %.

Synthetic strategy for **1**:

$[Dy^{III}L^{N6}(CH_3CO_2)_2](CH_3CO_2)\cdot 9H_2O$ (0.05 mmol, 44 mg) was dissolved in 5 ml of DCM giving a yellow solution. To this solution, the sodium salt of 2,4-di-tert-butylphenol, 2,4-di-tBu-PhONa (0.2 mmol, 46 mg) was added resulting in a dark red suspension. The reaction mixture was stirred at room temperature for 24 h. Then, $NaPF_6$ (0.05 mmol, 9 mg) was dissolved in 5 ml of

deionized H_2O and was added to the reaction mixture creating two layers. This mixture was refluxed at 100 ^oC for 1 h. Then the dark red DCM layer was separated from the colorless water layer and filtered. Red-brown prism-like crystals of $[Dy^{III}(L^{N6})(2,4\text{-di-}^tBu\text{-PhO})_2](PF_6)\cdot CH_2Cl_2$ (**1**$\cdot CH_2Cl_2$) were isolated by slowly diffusing Et_2O into the DCM solution. Elemental Anal. calcd (found) for **1**: C 52.02 (51.65), H 5.99 (5.67), N 7.14 (7.54) %.

Synthetic strategy for **2**:

$[Dy^{III}L^{N6}(CH_3CO_2)_2](CH_3CO_2)\cdot 9H_2O$ (0.05 mmol, 44 mg) was dissolved in 5 ml of DCM giving a yellow solution. To this solution, Ph_3SiOH (0.2 mmol, 55 mg) was added, resulting in a dark yellow suspension. The reaction mixture was stirred at room temperature for 24 h. Then $NaPF_6$ (0.05 mmol, 9 mg) was dissolved in 5 ml of deionized H_2O and was added to the reaction mixture creating two layers. This mixture was refluxed at 100 ^oC for 1 h. Then the dark yellow DCM layer was separated from the water layer and filtered. Yellow block-like crystals of $[Dy^{III}(L^{N6})(Ph_3SiO)_2](PF_6)$ (**2**) were isolated by slowly diffusing Et_2O into the DCM solution. Elemental Anal. calcd (found) for **2**$\cdot 0.3H_2O$: C 56.26 (56.56), H 4.61 (4.45), N 6.79 (6.46) %.

Synthetic strategy for **3**:

$[Dy^{III}L^{N6}(CH_3CO_2)_2](CH_3CO_2)\cdot 9H_2O$ (0.05 mmol, 44 mg) was dissolved in a mixture of DCM and deionized H_2O solution (1:1, 10 ml). To this suspension, Ph_3SiOH (0.2 mmol, 55 mg) was added. The reaction mixture was stirred at room temperature for 24 h. Then, sodium tetraphenylborate ($NaBPh_4$) (0.05 mmol, 17 mg) was transferred into the reaction mixture. This mixture was refluxed at 100 ^oC for 1 h. Then the dark yellow DCM layer was separated from the water layer and filtered. Gold prism-like crystals of $[Dy^{III}(L^{N6})(Ph_3SiO)_2](BPh_4)$ (**3**) were isolated by slowly diffusing Et_2O into the DCM solution. Elemental Anal. calcd (found) for **3**$\cdot 0.7H_2O$: C 69.38 (69.27), H 5.5 (5.39), N 5.92 (5.95) %.

Crystallographic details

All structures were solved using ShelxT (*SHELXT*: Sheldrick, G. M. (2015). *Acta Cryst.* A71, 3-8.) and refined using ShelxL (Sheldrick, G.M. (2015). Acta Cryst. C71, 3-8) within the program Olex2 (Dolomanov, O.V., Bourhis, L.J., Gildea, R.J, Howard, J.A.K. & Puschmann, H. (2009), J. Appl. Cryst. 42, 339-341). All non-hydrogen atoms were refined with anistropic atomic displacement parameters (ADPs) with hydrogens placed in geometrically calculated positions and included as part of a riding model or as a rigid rotor for Me groups. In **1** disorder in a DCM molecule was modelled with one chlorine atom over two partially occupied sites with occupancy 0.78:0.22. **2** crystallises with channels running parallel to the *c*-axis which contain poorly defined solvent. PLATON SQUEEZE (Spek, A. L. (2015). *Acta Cryst.* C**71**, 9-18) was used to calculated and account for this electron density, giving a solvent accessible volume of 5563 Å^3 (~20% of the unit cell volume) containing 1537 electrons or ~85 electrons per formula unit. Additionally one Ph ring of one silanol showed disorder and was modelled over two partially occupied sites with occupancies 0.581(9) and 0.419(9) and atom C45 common to both orientations. Further details are given in the CIFs and the tables below.

Table S1. Crystallographic data for complexes **$[Dy^{III}L^{N6}(CH_3CO_2)_2](CH_3CO_2)\cdot 9H_2O$**, **1**, **2** and **3**. [2]

		1	2	3
Formula	$C_{28}H_{53}DyN_6O_{15}$	$C_{51}H_{70}DyN_6O_2Cl_2F_6P$	$C_{58}H_{56}DyN_6O_2Si_2F_6P$	$C_{82}H_{76}DyN_6O_2Si_2B$
M_W	876.26	1177.50	1232.73	1406.97
Crystal System	Triclinic	Triclinic	Trigonal	Monoclinic
Space group	*P*-1	*P*-1	*R*-3	$P2_1/n$
a/Å	10.9695 (7)	13.9218 (7)	44.406 (3)	15.8869 (6)
b/Å	12.5118 (8)	14.0486 (7)		28.6320 (14)
c/Å	15.0704 (9)	14.4128 (7)	17.5772 (9)	16.2142 (6)
$\alpha/^o$	85.285 (2)	85.453 (2)	90	90
$\beta/^o$	85.584 (2)	77.297 (2)	90	111.943 (1)
$\gamma/^o$	64.963 (2)	77.118 (2)	120	90
V/Å^3	1865.6 (2)	2679.3 (2)	30017 (4)	6841.1 (5)
Z	2	2	18	4
T/K	150	150	150	150
λ/Å	0.71073	0.71073	0.71073	0.71073
D_c/g cm^{-3}	1.560	1.460	1.228	1.366
μ(Mo-K_α)/ mm^{-1}	2.08	1.59	1.24	1.18
Meas./indep.(R_{int}) refl.	27839/9178(0.048)	38585/13240(0.026)	68206/11785(0.039)	62328/15673(0.037)
Obs. refl. [$I>2\sigma(I)$]	8631	12107	10013	13114
$wR(F^2)$	0.070	0.068	0.190	0.078
$R[F^2 > 2s(F^2)]$	0.027	0.026	0.082	0.031
S	1.04	1.03	1.04	1.04
$\Delta\rho_{max,min}$/ eÅ^{-3}	1.68, -0.80	1.39,-0.85	3.01,-1.50	0.93,-0.54

Table S2. Selected bond distances and angles for complex **1** (Å, º). [2]

Dy1—O1A	2.1456 (14)	N1—Dy1—N2	60.16 (5)
Dy1—O1B	2.1303 (14)	N3—Dy1—N1	123.08 (5)
Dy1—N1	2.6089 (17)	N3—Dy1—N2	62.93 (6)
Dy1—N2	2.6383 (17)	N3—Dy1—N4	60.87 (5)
Dy1—N3	2.5892 (17)	N4—Dy1—N1	158.72 (6)
Dy1—N4	2.6000 (17)	N4—Dy1—N2	118.58 (5)
Dy1—N5	2.5878 (17)	N5—Dy1—N1	117.17 (5)
Dy1—N6	2.5722 (17)	N5—Dy1—N2	172.07 (5)
O1A—Dy1—N1	78.67 (6)	N5—Dy1—N3	119.38 (6)
O1A—Dy1—N2	83.36 (6)	N5—Dy1—N4	60.79 (5)
O1A—Dy1—N3	96.36 (6)	N6—Dy1—N1	60.50 (6)
O1A—Dy1—N4	80.10 (5)	N6—Dy1—N2	117.28 (6)
O1A—Dy1—N5	88.79 (6)	N6—Dy1—N3	160.25 (6)
O1A—Dy1—N6	103.32 (6)	N6—Dy1—N4	124.04 (5)
O1B—Dy1—O1A	176.54 (5)	N6—Dy1—N5	63.45 (6)
O1B—Dy1—N1	104.31 (6)	O1B—Dy1—N4	96.95 (6)
O1B—Dy1—N2	96.55 (6)	O1B—Dy1—N5	91.36 (6)
O1B—Dy1—N3	80.56 (5)	O1B—Dy1—N6	79.82 (6)

Table S3. Selected bond distances and angles for complex **2** (Å, º). [2]

Dy1—O1	2.153 (7)	O2—Dy1—N4	79.7 (2)
Dy1—O2	2.163 (6)	O2—Dy1—N5	98.9 (2)
Dy1—N1	2.616 (6)	O2—Dy1—N6	87.3 (2)
Dy1—N2	2.551 (6)	N1—Dy1—N3	117.1 (2)
Dy1—N3	2.642 (6)	N1—Dy1—N4	159.1 (2)
Dy1—N4	2.623 (6)	N1—Dy1—N6	60.9 (2)
Dy1—N5	2.583 (7)	N2—Dy1—N1	60.79 (19)
Dy1—N6	2.621 (6)	N2—Dy1—N3	62.8 (2)
O1—Dy1—O2	179.8 (2)	N2—Dy1—N4	122.1 (2)
O1—Dy1—N1	100.6 (2)	N2—Dy1—N5	160.9 (2)
O1—Dy1—N2	80.0 (2)	N2—Dy1—N6	118.4 (2)
O1—Dy1—N3	94.9 (2)	N4—Dy1—N3	59.5 (2)
O1—Dy1—N4	100.2 (2)	N5—Dy1—N1	124.4 (2)
O1—Dy1—N5	81.0 (2)	N5—Dy1—N3	118.2 (2)
O1—Dy1—N6	92.6 (3)	N5—Dy1—N4	60.8 (2)
O2—Dy1—N1	79.5 (2)	N5—Dy1—N6	63.4 (3)
O2—Dy1—N2	100.1 (2)	N6—Dy1—N3	172.5 (2)
O2—Dy1—N3	85.2 (2)	N6—Dy1—N4	119.4 (2)

Table S4. Selected bond distances and angles for complex **3** (Å, º). [2]

Dy1—O1	2.1425 (16)	O2—Dy1—N5	85.24 (6)
Dy1—O2	2.1514 (16)	O2—Dy1—N6	103.13 (6)
Dy1—N1	2.627 (2)	N1—Dy1—N2	59.97 (7)
Dy1—N2	2.635 (2)	N3—Dy1—N1	121.77 (7)
Dy1—N3	2.610 (2)	N3—Dy1—N2	62.71 (7)
Dy1—N4	2.6057 (18)	N3—Dy1—N5	119.34 (6)
Dy1—N5	2.6242 (19)	N3—Dy1—N6	159.02 (6)
Dy1—N6	2.6188 (19)	N4—Dy1—N1	166.12 (6)
O1—Dy1—O2	176.13 (6)	N4—Dy1—N2	117.89 (6)
O1—Dy1—N1	97.45 (6)	N4—Dy1—N3	60.72 (6)
O1—Dy1—N2	97.45 (7)	N4—Dy1—N5	60.44 (6)
O1—Dy1—N3	80.26 (6)	N4—Dy1—N6	122.78 (6)
O1—Dy1—N4	96.42 (6)	N5—Dy1—N1	118.89 (6)
O1—Dy1—N5	92.73 (6)	N5—Dy1—N2	169.82 (7)
O1—Dy1—N6	78.79 (6)	N6—Dy1—N1	60.60 (7)
O2—Dy1—N1	86.41 (6)	N6—Dy1—N2	119.28 (7)
O2—Dy1—N2	84.58 (7)	N6—Dy1—N5	62.88 (6)
O2—Dy1—N3	97.85 (6)	O2—Dy1—N4	79.71 (6)

Table S5. Shape measures of complex **1**. The lowest CShMs value, is highlighted.[3]

	Dy	Symmetry	Ideal polyhedron
OP-8	30.698	D_{8h}	Octagon
HPY-8	22.260	C_{7v}	Heptagonal pyramid
**HBPY-8**	**2.472**	$\boldsymbol{D_{6h}}$	**Hexagonal bipyramid**
CU-8	10.589	O_h	Cube
SAPR-8	15.282	D_{4d}	Square antiprism
TDD-8	13.470	D_{2d}	Triangular dodecahedron
JGBF-8	4.817	D_{2d}	Johnson gyrobifastigium J26
JBTPR-8	13.704	C_{2v}	Biaugmented trigonal prism J50
BTPR-8	13.597	C_{2v}	Biaugmented trigonal prism
JSD-8	13.223	D_{2d}	Snub diphenoid J84
TT-8	11.325	T_d	Triakis tetrahedron
ETBPY-8	20.156	D_{3h}	Elongated trigonal bipyramid

Table S6. Shape measures of complex **2**. The lowest CShMs value, is highlighted.[3]

	Dy	Symmetry	Ideal polyhedron
OP-8	31.775	D_{8h}	Octagon
HPY-8	22.953	C_{7v}	Heptagonal pyramid
**HBPY-8**	**2.271**	$\boldsymbol{D_{6h}}$	**Hexagonal bipyramid**
CU-8	10.304	O_h	Cube
SAPR-8	15.565	D_{4d}	Square antiprism
TDD-8	13.574	D_{2d}	Triangular dodecahedron
JGBF-8	4.983	D_{2d}	Johnson gyrobifastigium J26
JBTPR-8	14.210	C_{2v}	Biaugmented trigonal prism J50
BTPR-8	14.080	C_{2v}	Biaugmented trigonal prism
JSD-8	14.110	D_{2d}	Snub diphenoid J84
TT-8	10.982	T_d	Triakis tetrahedron
ETBPY-8	20.803	D_{3h}	Elongated trigonal bipyramid

Table S7. Shape measures of complex **3**. The lowest CShMs value, is highlighted.[3]

	Dy	Symmetry	Ideal polyhedron
OP-8	31.062	D_{8h}	Octagon
HPY-8	22.24	C_{7v}	Heptagonal pyramid
**HBPY-8**	**2.163**	$\boldsymbol{D_{6h}}$	**Hexagonal bipyramid**
CU-8	10.442	O_h	Cube
SAPR-8	16.653	D_{4d}	Square antiprism
TDD-8	13.991	D_{2d}	Triangular dodecahedron
JGBF-8	5.635	D_{2d}	Johnson gyrobifastigium J26
JBTPR-8	15.05	C_{2v}	Biaugmented trigonal prism J50
BTPR-8	14.787	C_{2v}	Biaugmented trigonal prism
JSD-8	14.946	D_{2d}	Snub diphenoid J84
TT-8	11.263	T_d	Triakis tetrahedron
ETBPY-8	20.693	D_{3h}	Elongated trigonal bipyramid

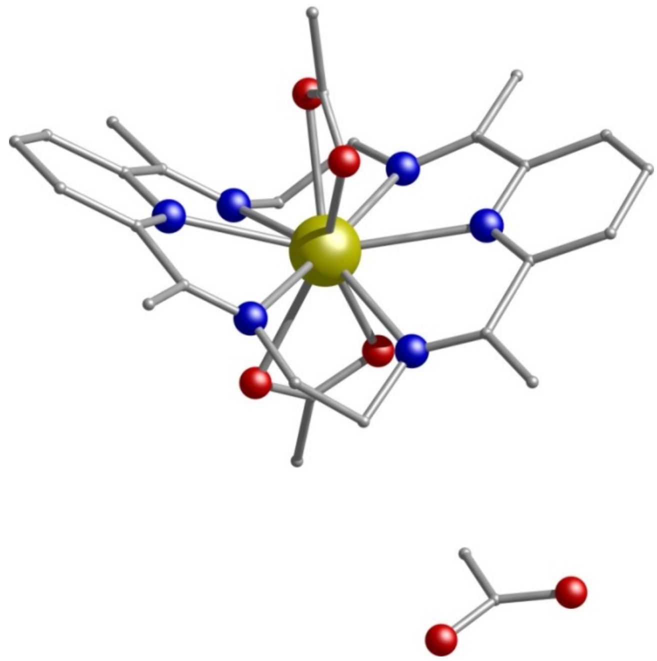

Figure S1. Molecular structure of **[Dy^{III}L^{N6}(CH$_3$CO$_2$)$_2$](CH$_3$CO$_2$)·9H$_2$O** with the acetate ligands chelating the axial positions of the dysprosium centre. Dy, gold; O, red; N, blue, C, grey. Hydrogen atoms and lattice solvent are omitted for clarity.

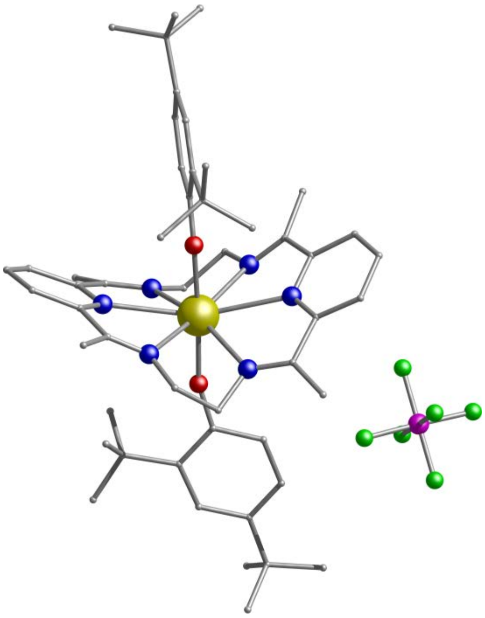

Figure S2. Molecular structure of **1** with 2,4-di-^{t}Bu-PhO^{-} as axial ligand. Dy, gold; O, red; N, blue, P, pink; F, green; C, grey. Hydrogen atoms and disorder components are omitted for clarity.

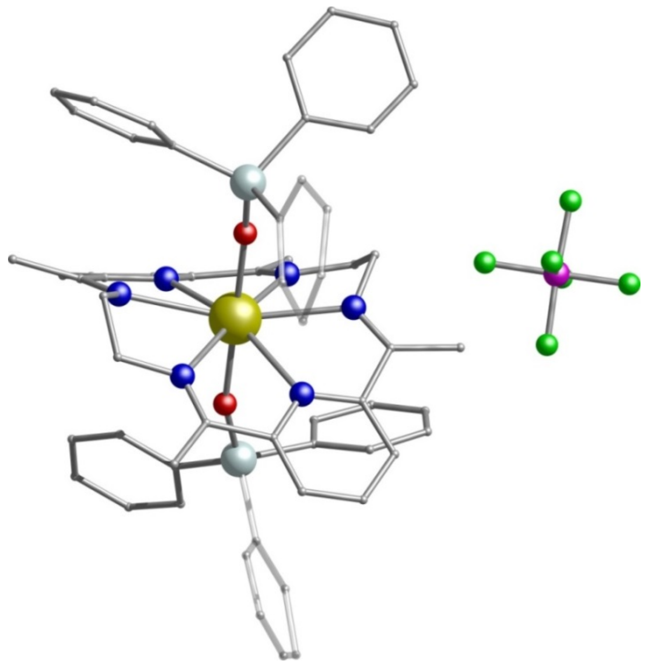

Figure S3. Molecular structure of **2** with Ph_3SiO^- as axial ligand. Dy, gold; O, red; N, blue, P, pink; F, green; Si, light turquoise; C, grey. Hydrogen atoms and disorder components are omitted for clarity.

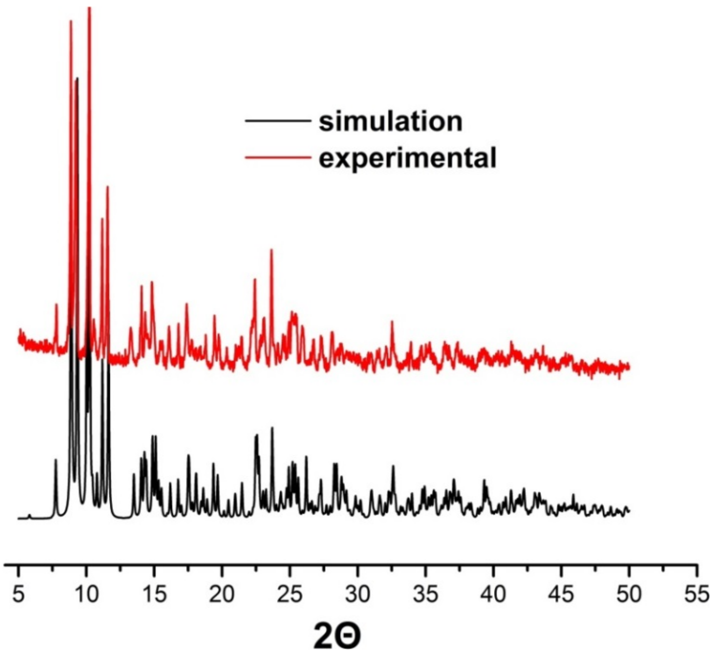

Figure S4. The powder X-ray diffraction pattern of $[Dy^{III}L^{N6}(CH_3CO_2)_2](CH_3CO_2)\cdot 9H_2O$. The black line represents the simulated powder X-ray diffraction pattern generated from single-crystal data collected at 150 K, and the red line represents the experimental data measured at ambient temperature.

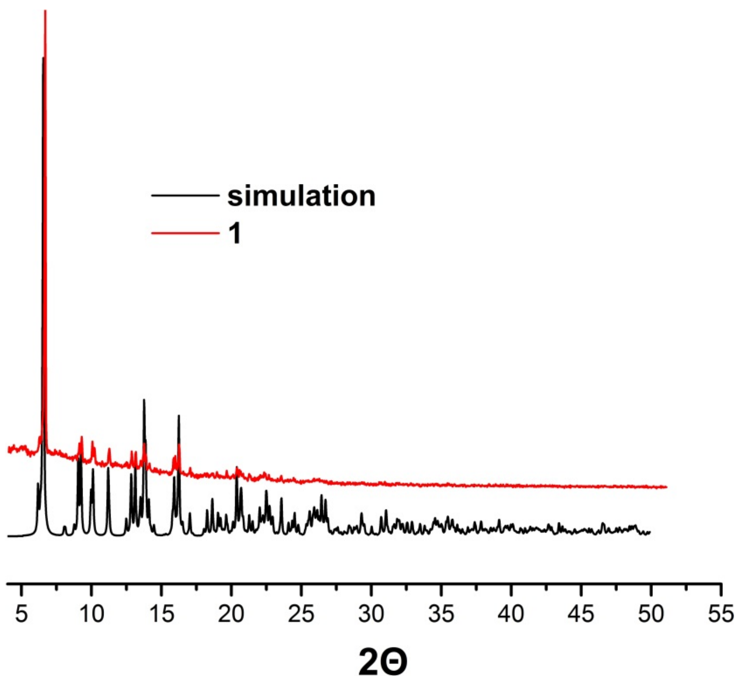

Figure S5. The powder X-ray diffraction pattern of **1**. The black line represents the simulated powder X-ray diffraction pattern generated from single-crystal data collected at 150 K, and the red line represents the experimental data measured at ambient temperature.

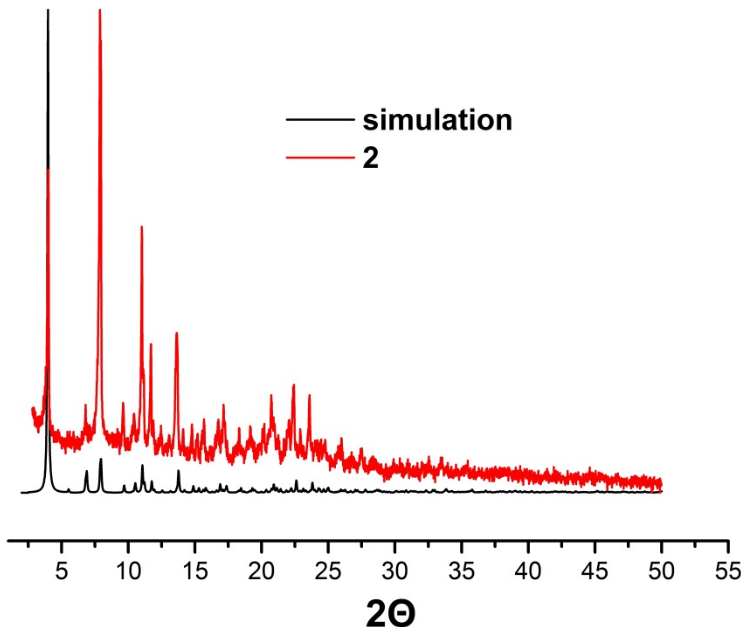

Figure S6. The powder X-ray diffraction pattern of **2**. The black line represents the simulated powder X-ray diffraction pattern generated from single-crystal data collected at 150 K, and the red line represents the experimental data measured at ambient temperature.

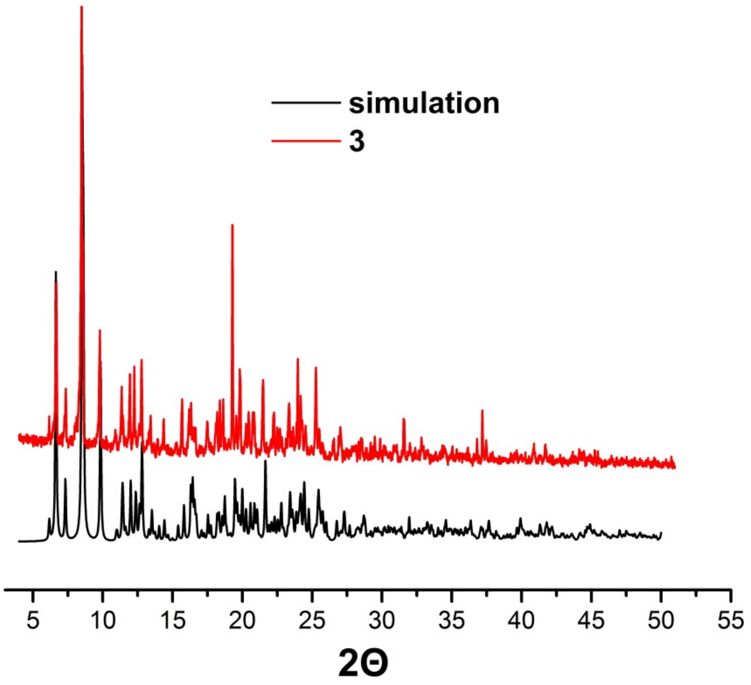

Figure S7. The powder X-ray diffraction pattern of **3**. The black line represents the simulated powder X-ray diffraction pattern generated from single-crystal data collected at 150 K, and the red line represents the experimental data measured at ambient temperature.

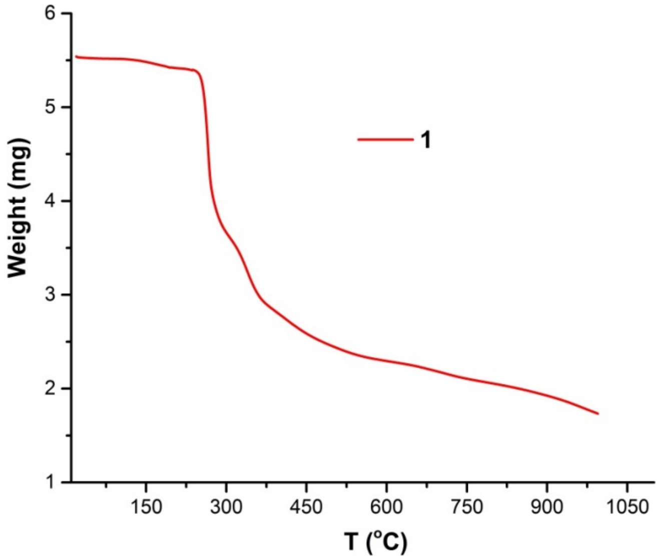

Figure S8. Thermogravimetric analysis for **1** under an argon atmosphere (10 K min^{-1}).

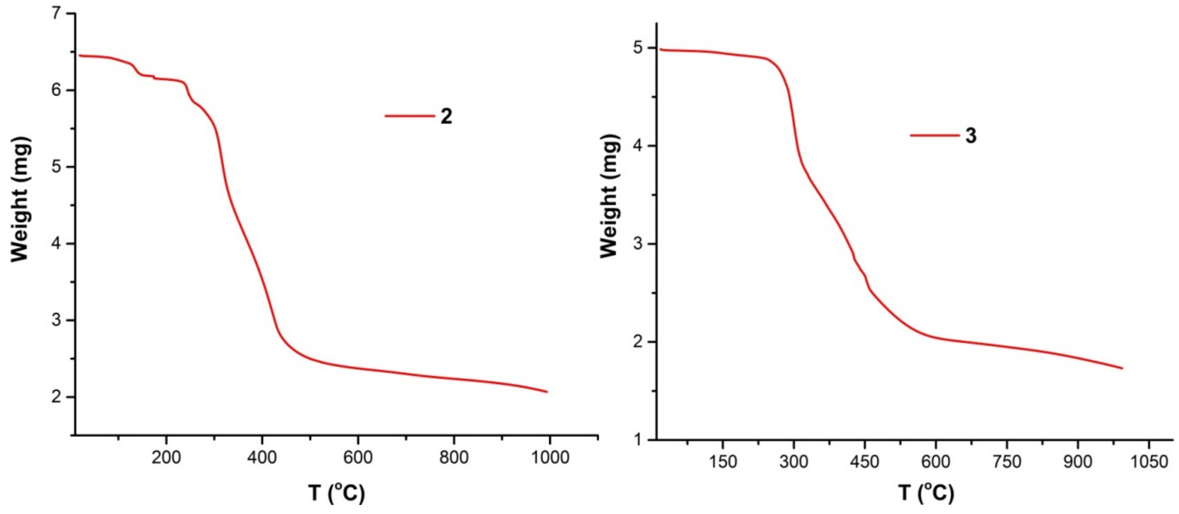

Figure S9. Thermogravimetric analysis for **2** (left) and **3** (right) under an argon atmosphere (10 K min^{-1}).

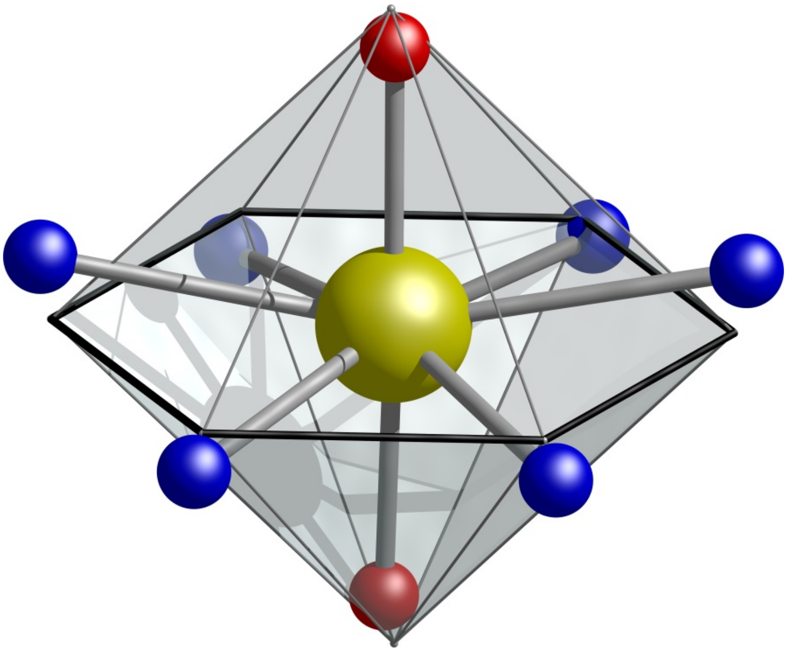

Figure S10. Comparison of the calculated (with SHAPE) [3] and experimental compressed hexagonal bipyramidal coordination sphere for the Dy(III) ion in **1**-**3** (shown for **2**). Dy, gold; N, dark blue; O, red.

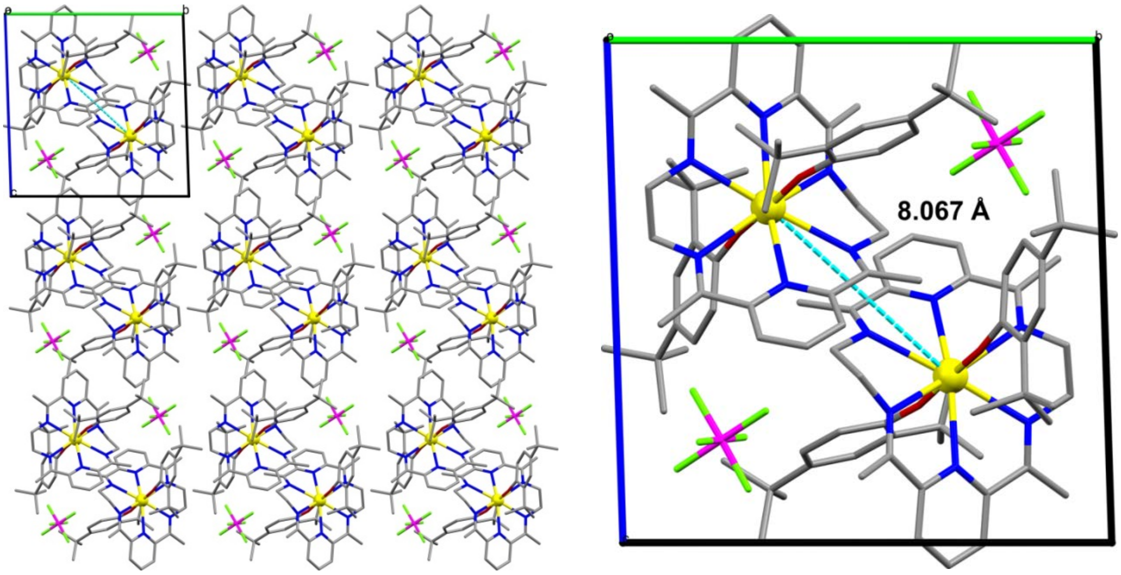

Figure S11. (Left) The crystal packing of **1** looking down the *a* axis, (Right) highlighting the shortest Dy⋯Dy distance in **1**. Hydrogen atoms, disorder components and solvent molecules are omitted for clarity. Dy, yellow; N, dark blue; O, red; P, pink; F, green; C, grey.

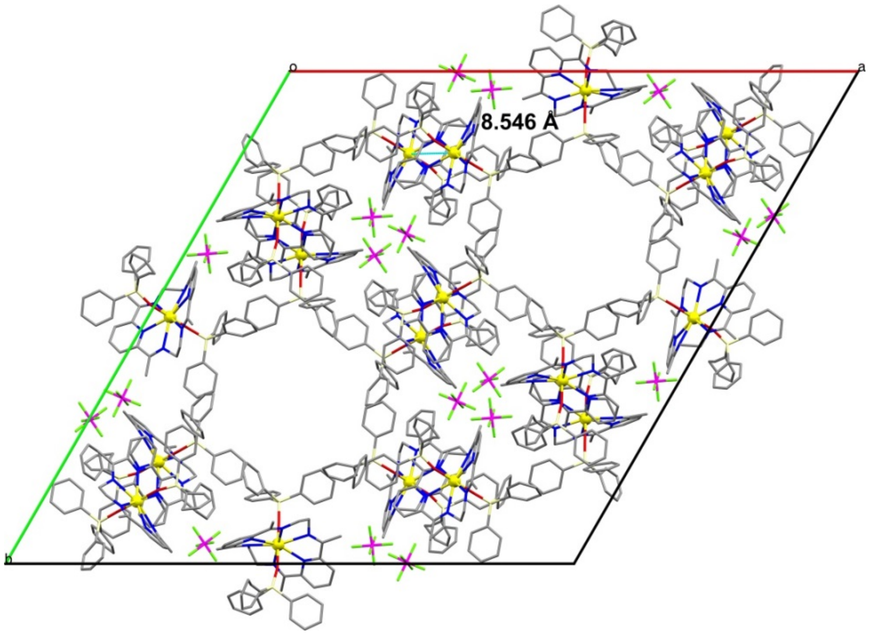

Figure S12. The crystal packing of **2** looking down the *c* axis. The shortest Dy⋯Dy distance is 8.546 Å for **2** . Hydrogen atoms are omitted for clarity. Solvent molecules occupying the channels parallel to the *c*-axis are not shown and were accounted for using SQUEEZE. Dy, yellow; N, dark blue; O, red; P, pink; F, green; Si, dark yellow; C, grey

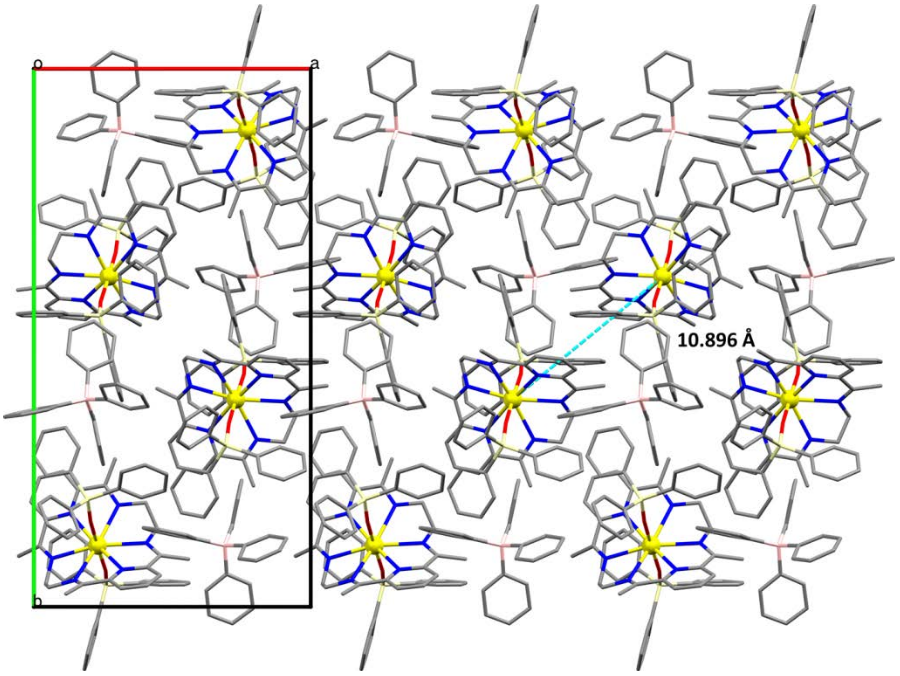

Figure S13. The crystal packing of **3** looking down the *c* axis with the shortest Dy···Dy distance of 10.896 Å. Hydrogen atoms and disorder components are omitted for clarity. Dy, yellow; N, dark blue; O, red; B, pink; Si, dark yellow; C, grey.

3. Magnetic characterization

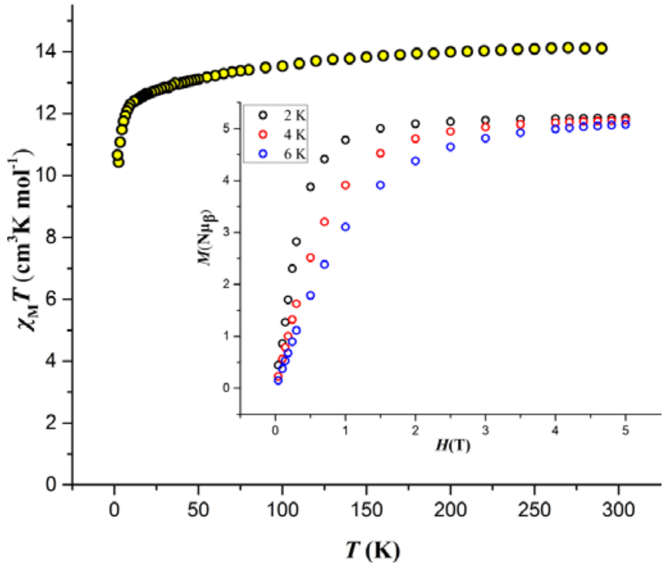

Figure S14. $\chi_M T$ vs. T data for **1** in a field of 1000 Oe from 290 – 2 K. Inset: Magnetisation vs. Field plot at temperatures 2, 4 and 6 K for **1** from 0.1-5 T.

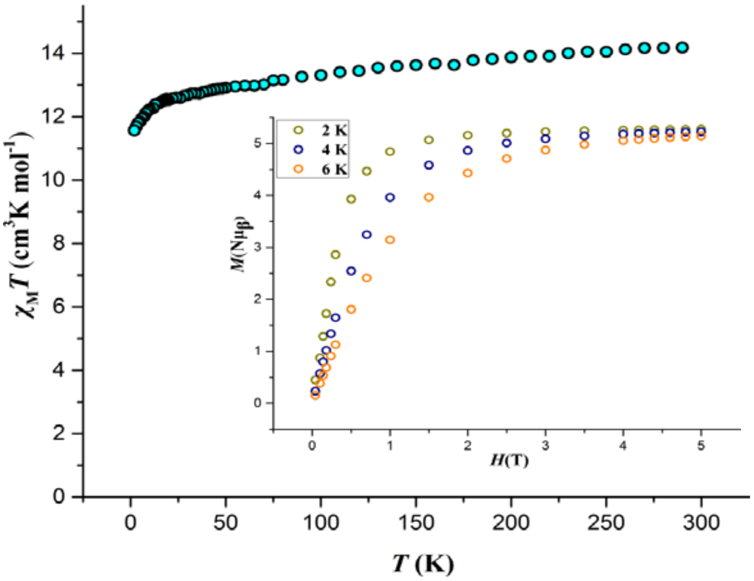

Figure S15. $\chi_M T$ vs. T data for **2** in a field of 1000 Oe from 290 – 2 K. Inset: Magnetisation vs. Field plot at temperatures 2, 4 and 6 K for **2** from 0.1-5 T.

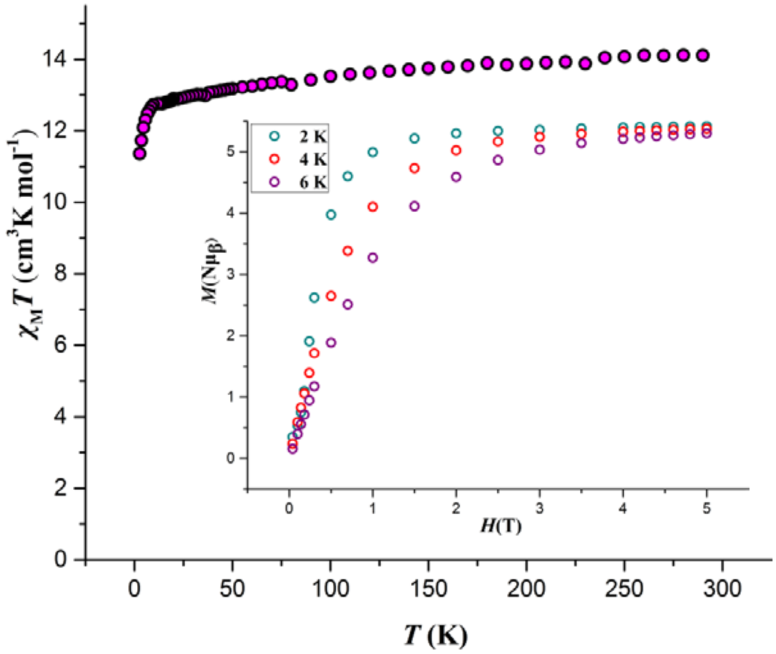

Figure S16. $\chi_M T$ vs. T data for **3** in a field of 1000 Oe from 290 – 2 K. Inset: Magnetisation vs. Field plot at temperatures 2, 4 and 6 K for **3** from 0.1-5 T.

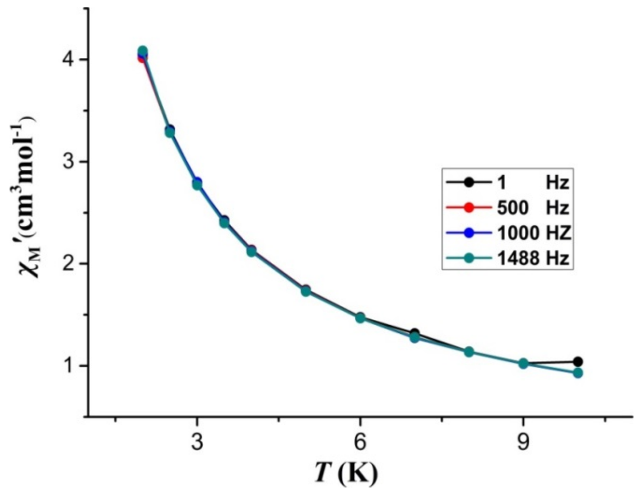

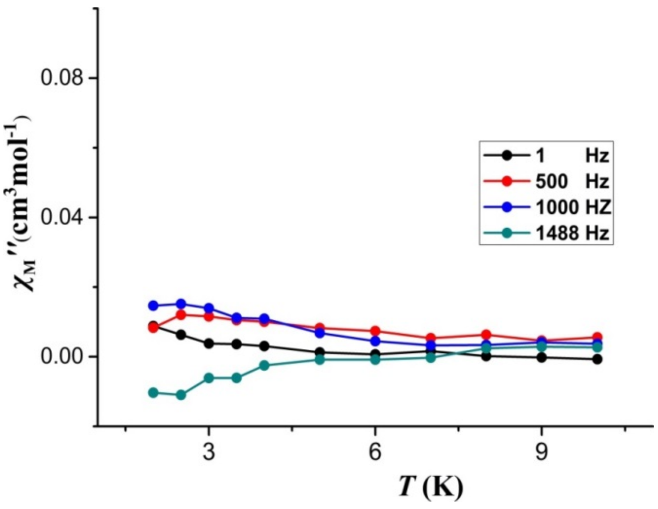

Figure S17. Temperature dependence of the in-phase, χ'_M (upper), and out-of-phase, χ''_M (lower) ac susceptibility, in zero dc field, for complex **$[Dy^{III}L^{N6}(CH_3CO_2)_2](CH_3CO_2)\cdot9H_2O$** showing no out-of-phase signal.

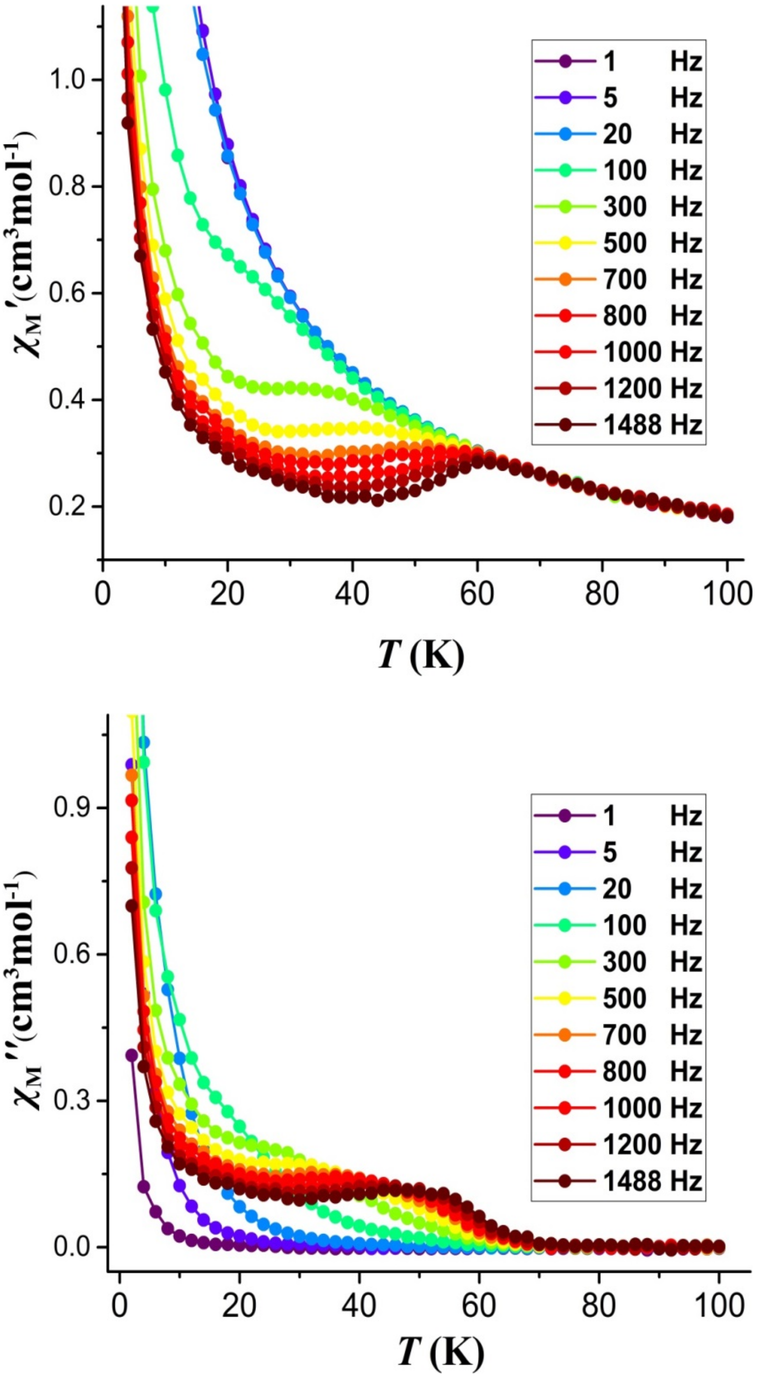

Figure S18. Temperature dependence of the in-phase, χ'_M (upper), and out-of-phase, χ''_M (lower) ac susceptibility, in zero dc field, for **1** with ac frequencies of 1−1488 Hz.

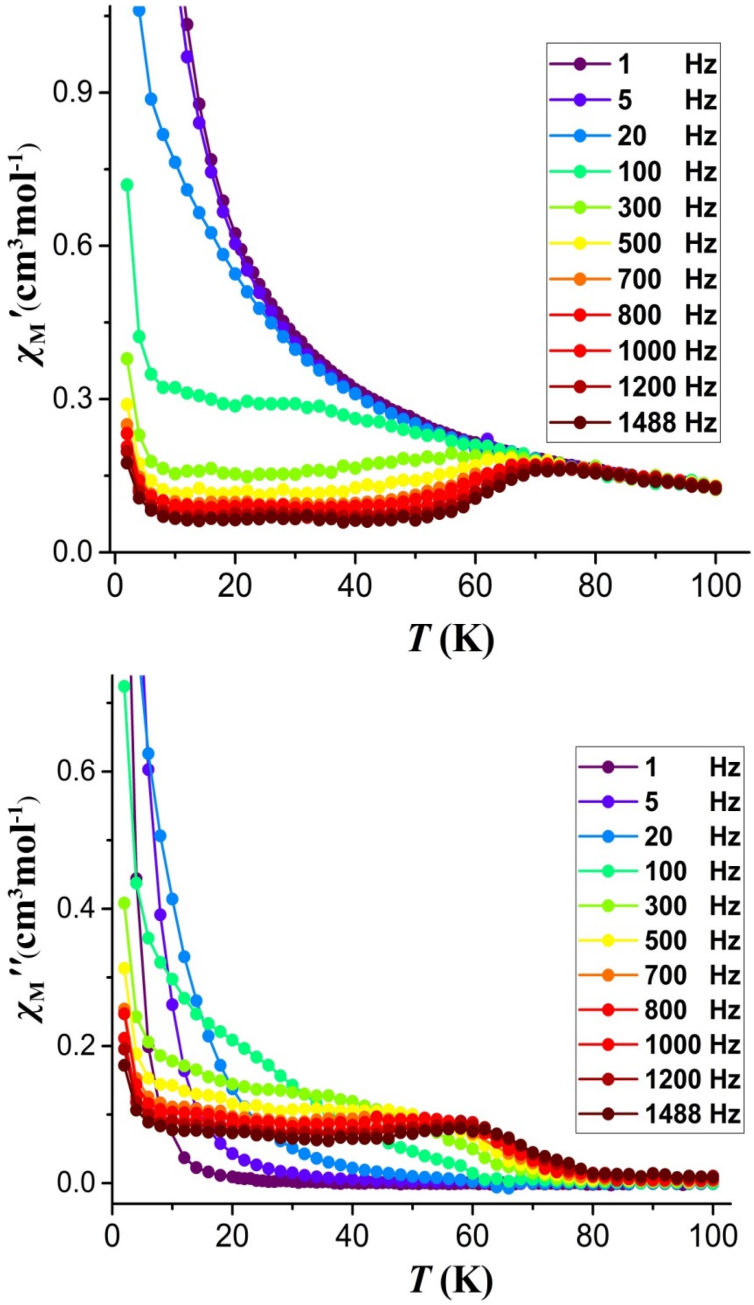

Figure S19. Temperature dependence of the in-phase, χ'_M (upper), and out-of-phase, χ''_M (lower) ac susceptibility, in zero dc field, for **2** with ac frequencies of 1−1488 Hz.

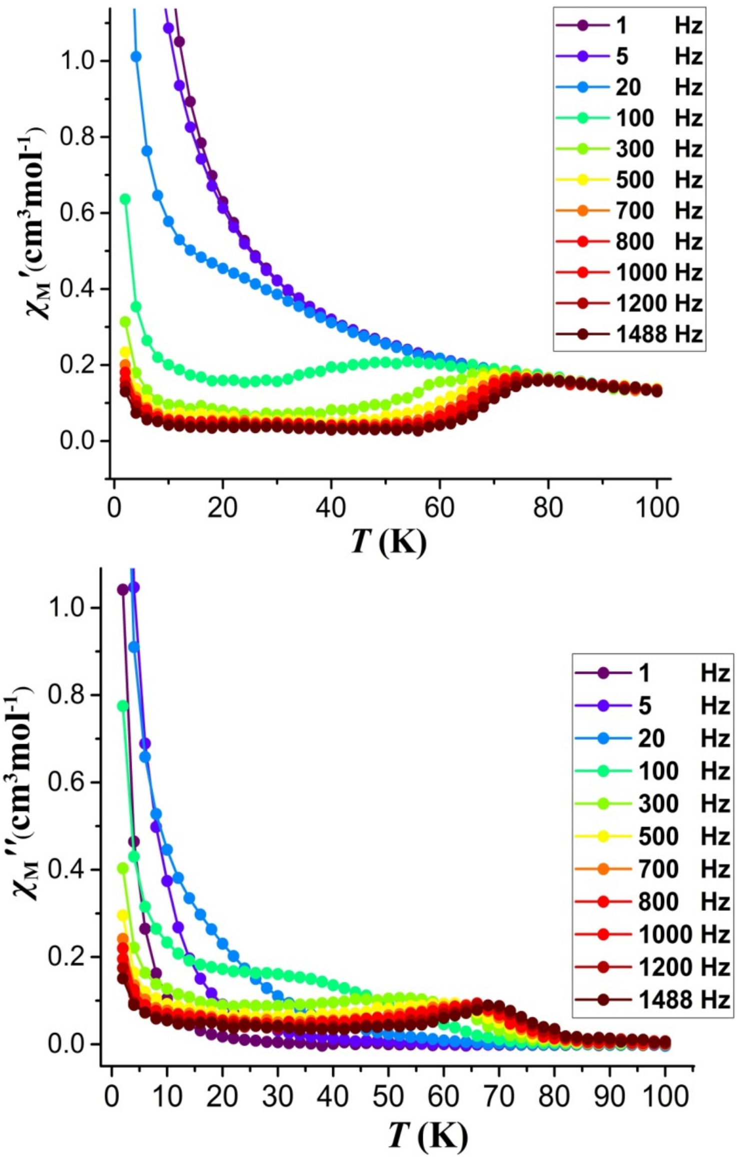

Figure S20. Temperature dependence of the in-phase, χ'_M (upper), and out-of-phase, χ''_M (lower) ac susceptibility, in zero dc field, for **3** with ac frequencies of 1−1488 Hz.

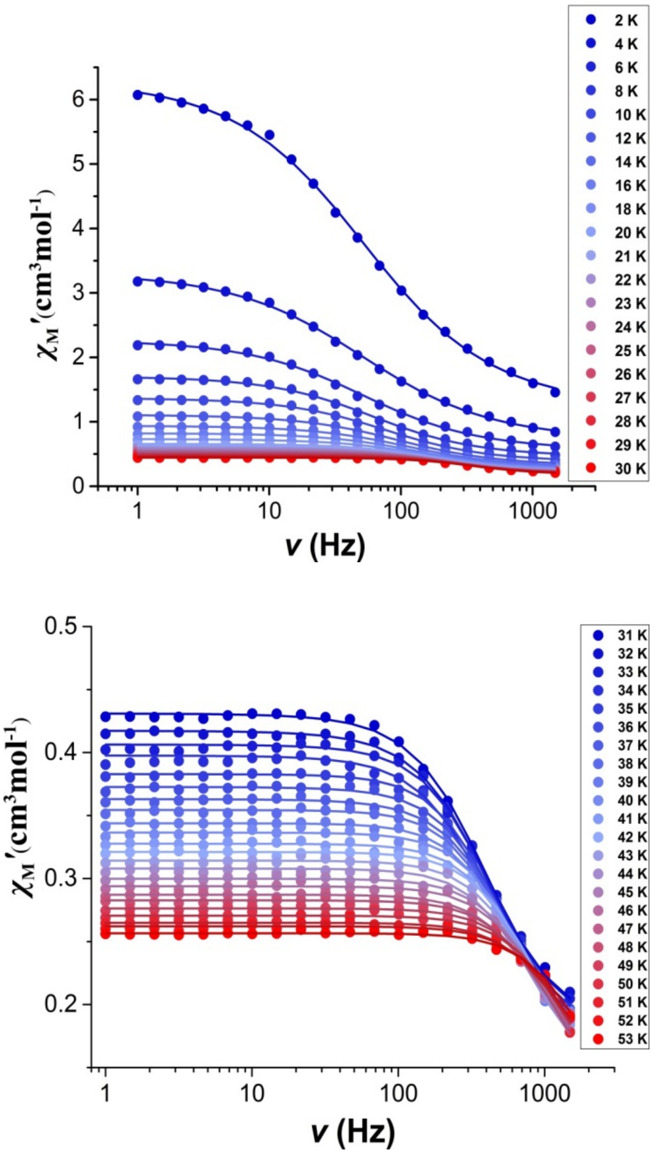

Figure S21. Frequency dependence of the in-phase ac susceptibility, in zero dc field from 2-30 (upper) and 31-53 K (lower) for **1**. The solid lines correspond to the best fit.

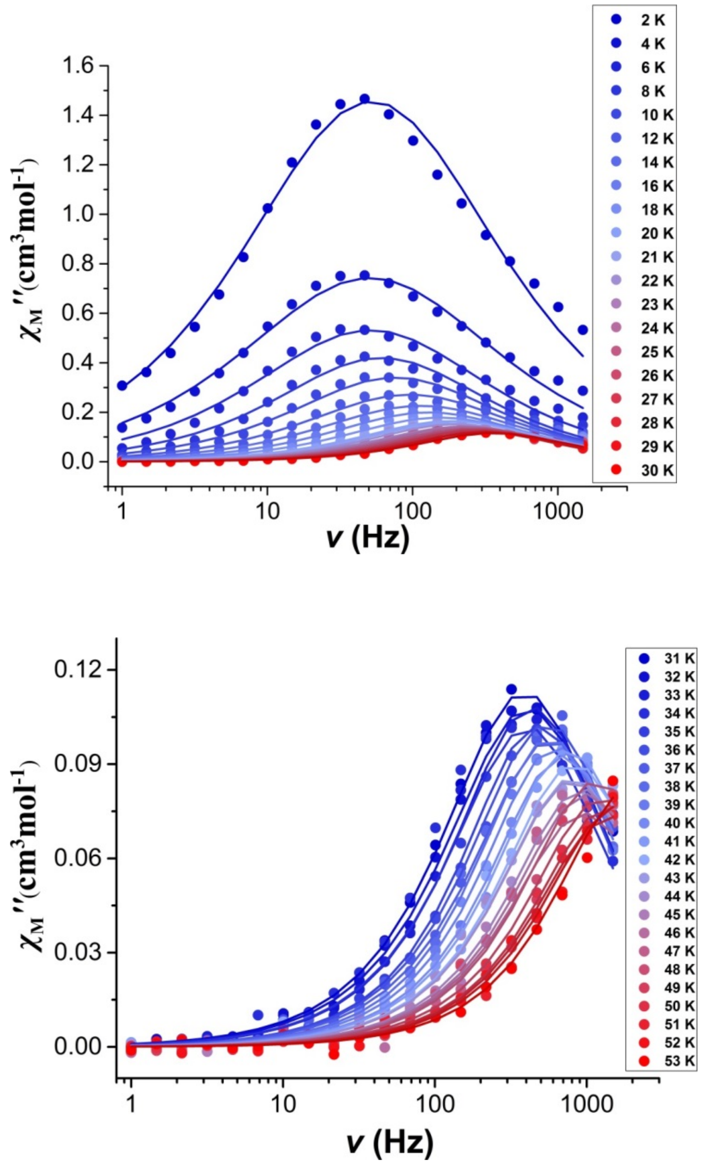

Figure S22. Frequency dependence of the out-of-phase ac susceptibility, in zero dc field from 2-30 (upper) and 31-53 K (lower) for **1**. The solid lines correspond to the best fit.

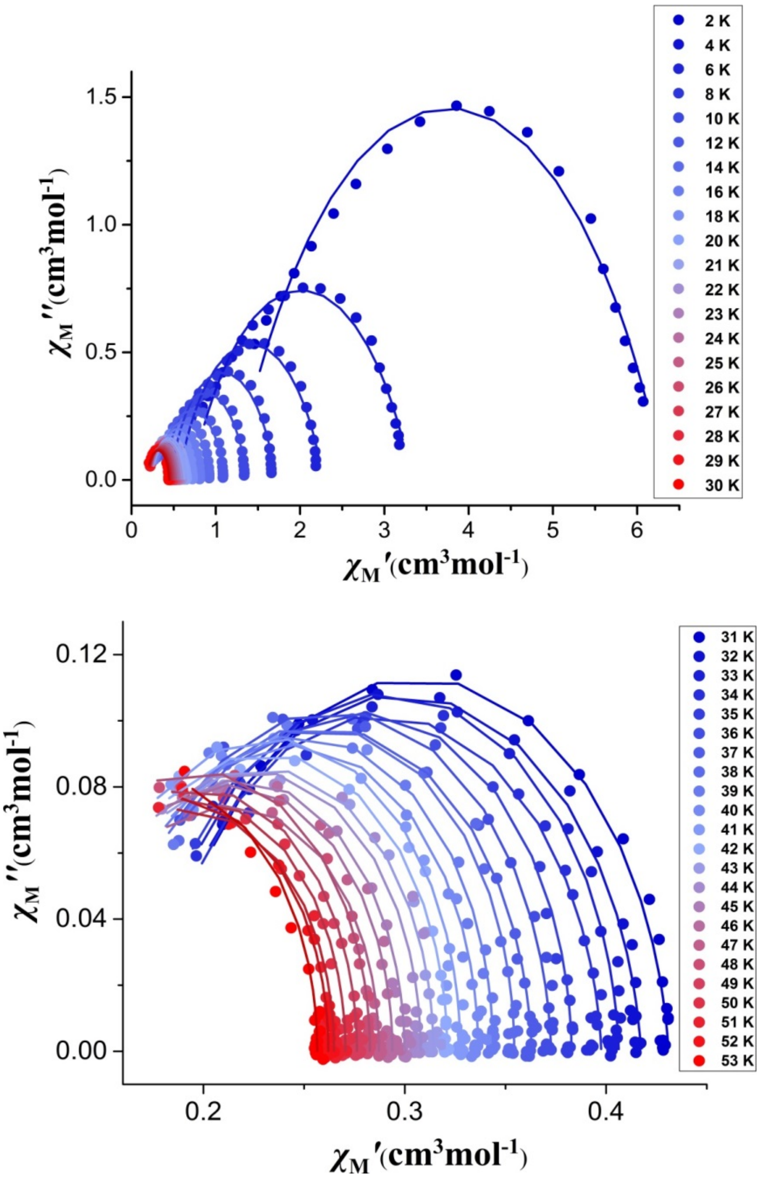

Figure S23. χ''_M vs χ'_M plot of the ac magnetic susceptibility, in zero dc field, from 2-30 (upper) and 31-53 K (lower) for **1**. The solid lines correspond to the best fit.

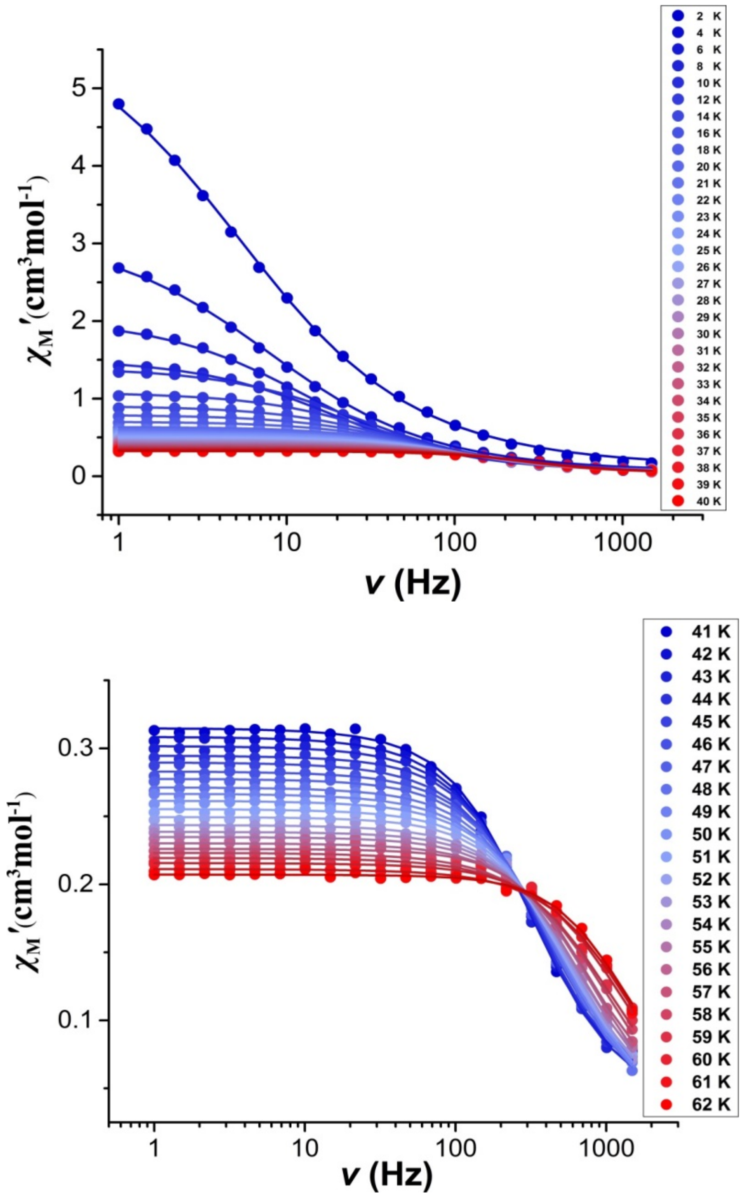

Figure S24. Frequency dependence of the in-phase ac susceptibility, in zero dc field from 2-40 (upper) and 41-62 K (lower) for **2**. The solid lines correspond to the best fit.

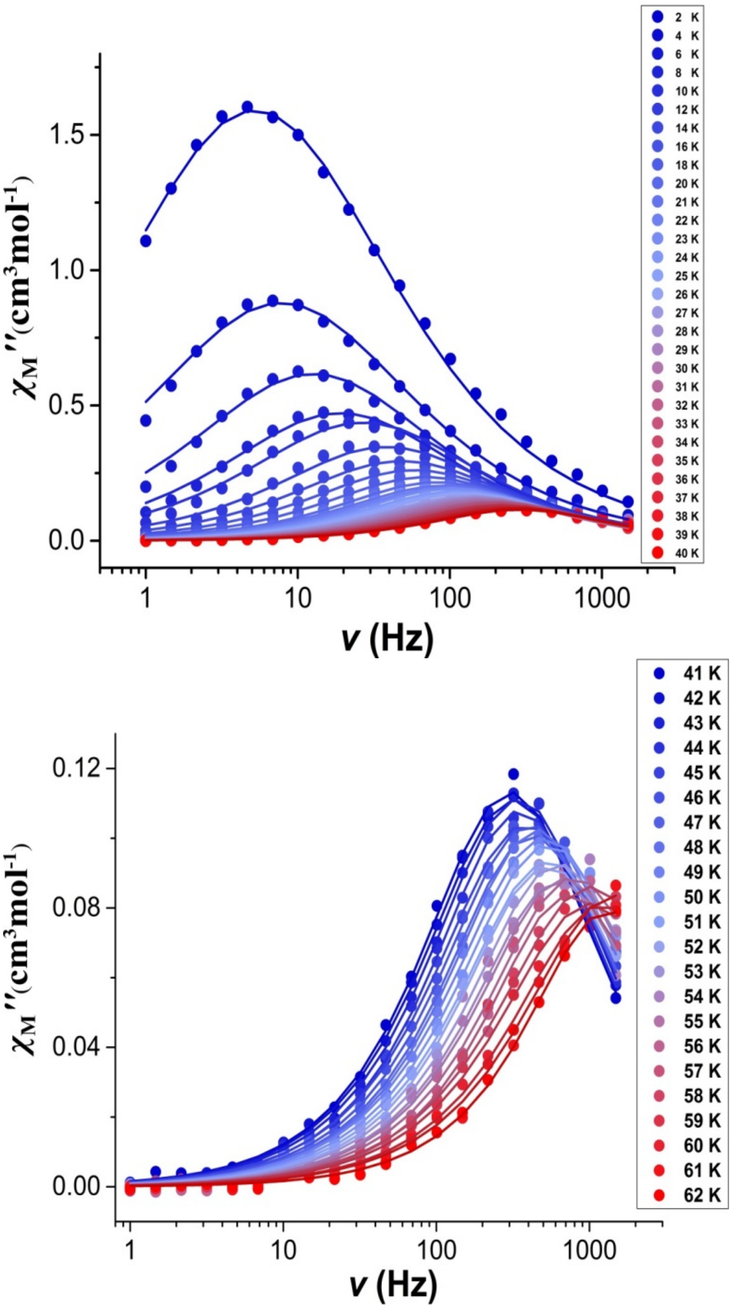

Figure S25. Frequency dependence of the out-of-phase ac susceptibility, in zero dc field from 2-40 (upper) and 41-62 K (lower) for **2**. The solid lines correspond to the best fit.

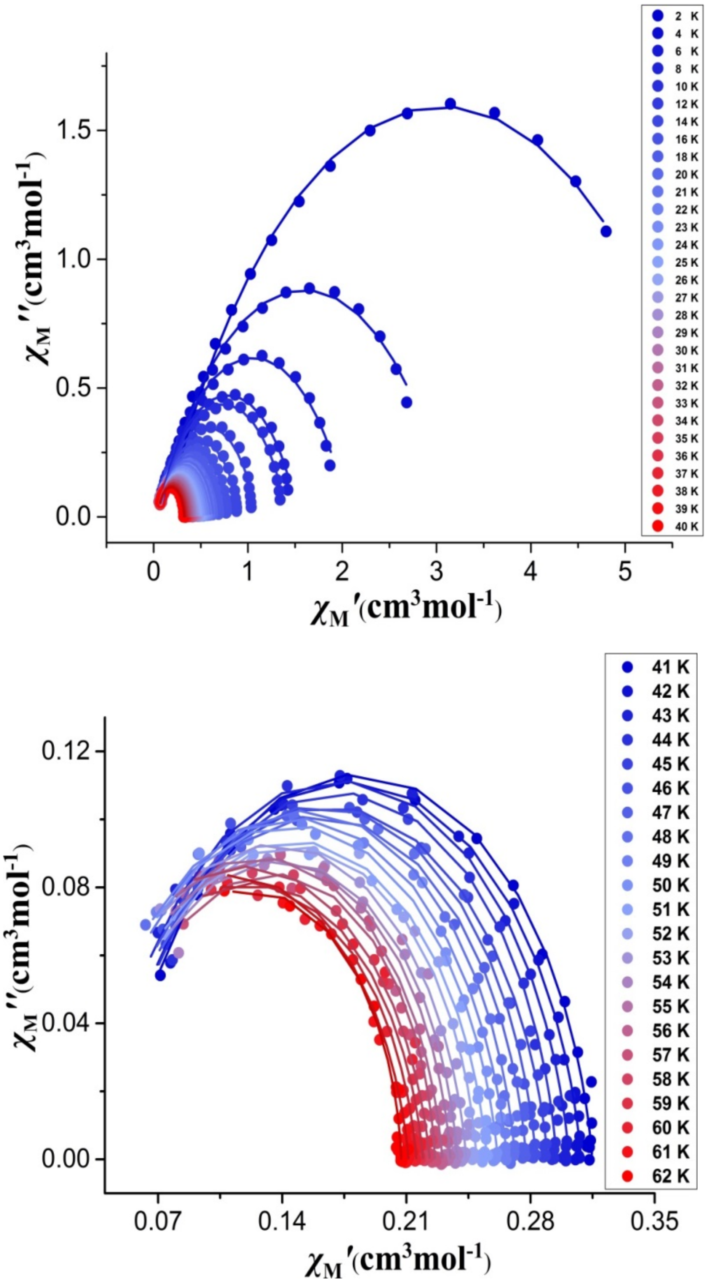

Figure S26. χ''_M vs χ'_M plot of the ac magnetic susceptibility, in zero dc field, from 2-40 (upper) and 41-62 K (lower) for **2**. The solid lines correspond to the best fit

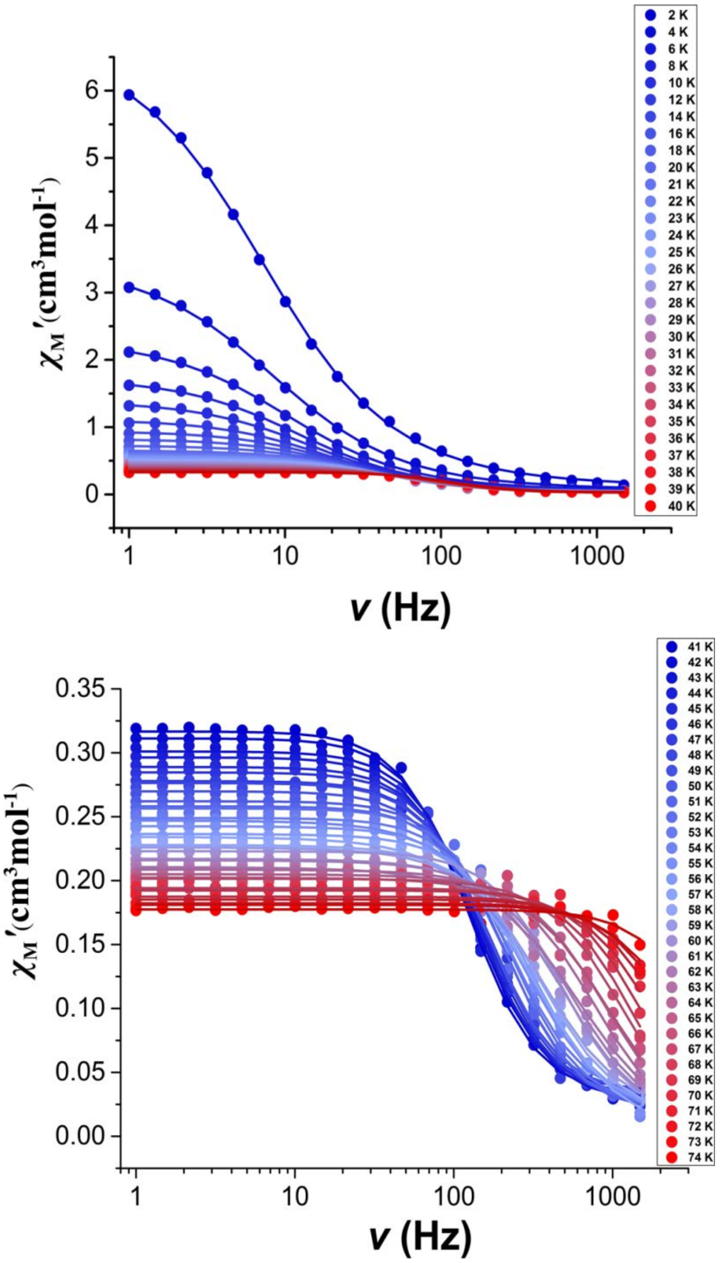

Figure S27. Frequency dependence of the in-phase ac susceptibility, in zero dc field from 2-40 (upper) and 41-74 K (lower) for **3**. The solid lines correspond to the best fit.

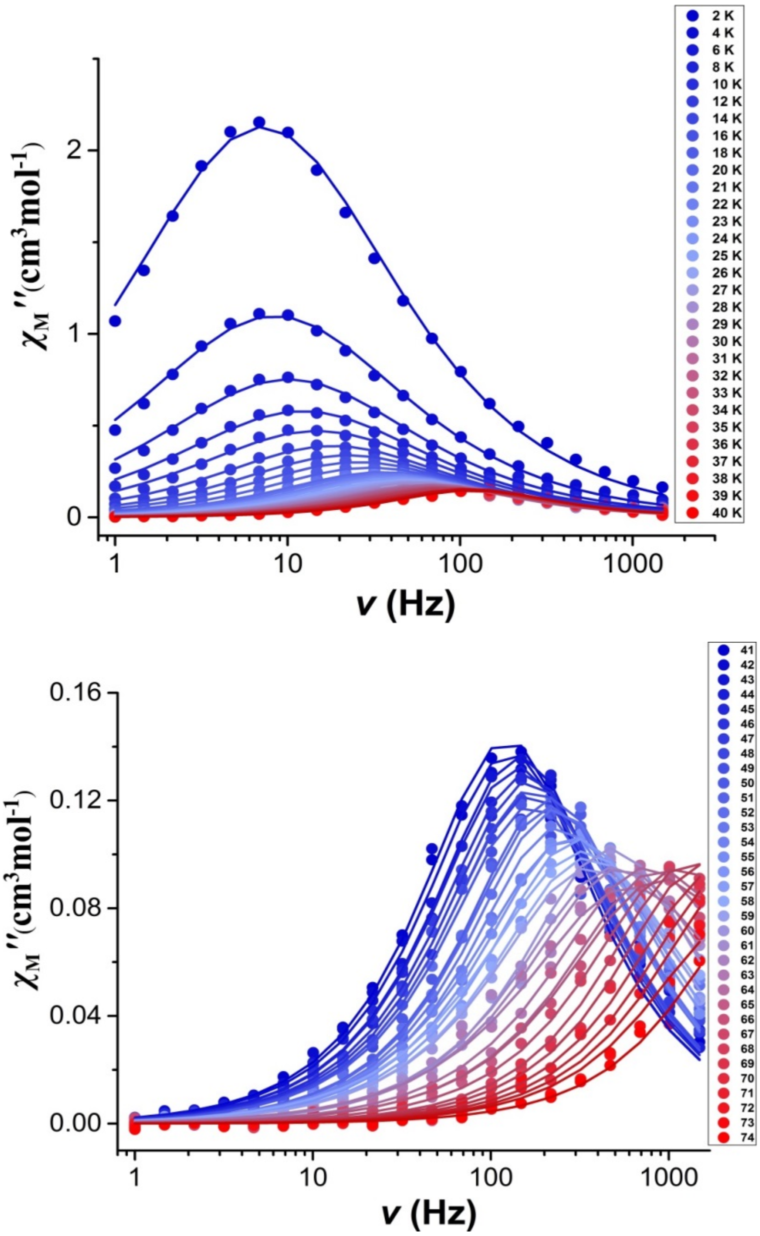

Figure S28. Frequency dependence of the out-of-phase ac susceptibility, in zero dc field from 2-40 (upper) and 41-74 K (lower) for **3**. The solid lines correspond to the best fit.

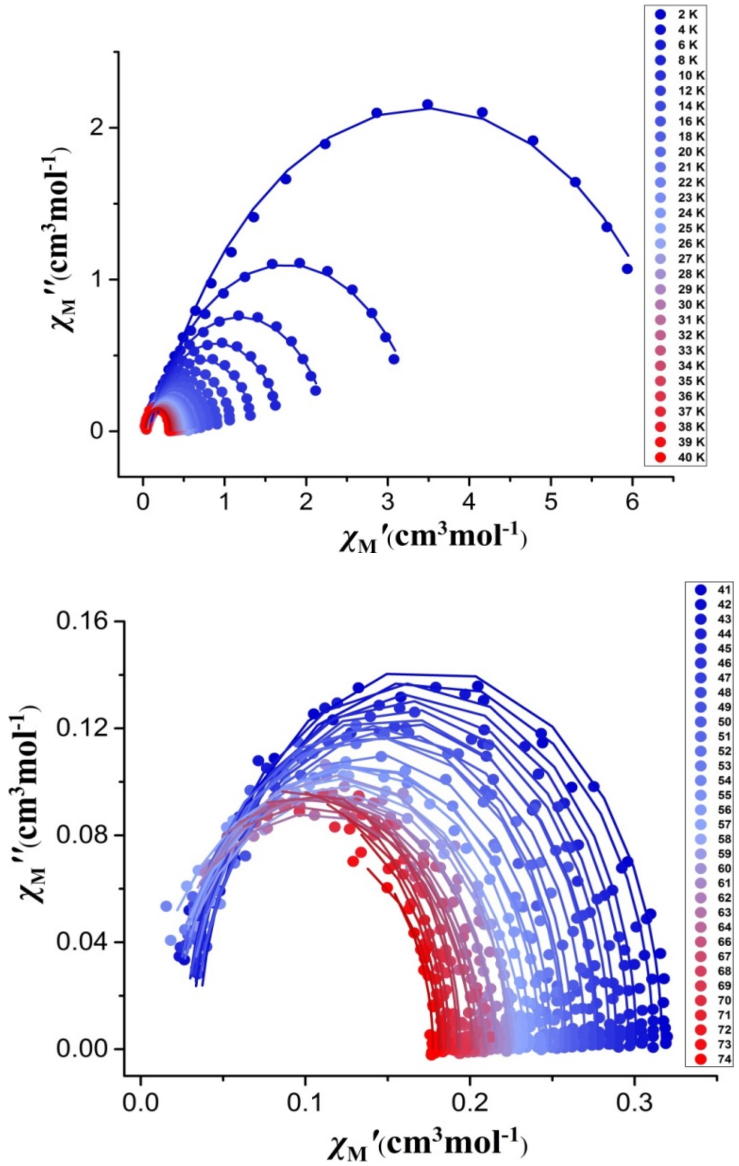

Figure S29. χ''_M vs χ'_M plot of the ac magnetic susceptibility, in zero dc field, from 2-40 (upper) and 41-74 K (lower) for **3**. The solid lines correspond to the best fit

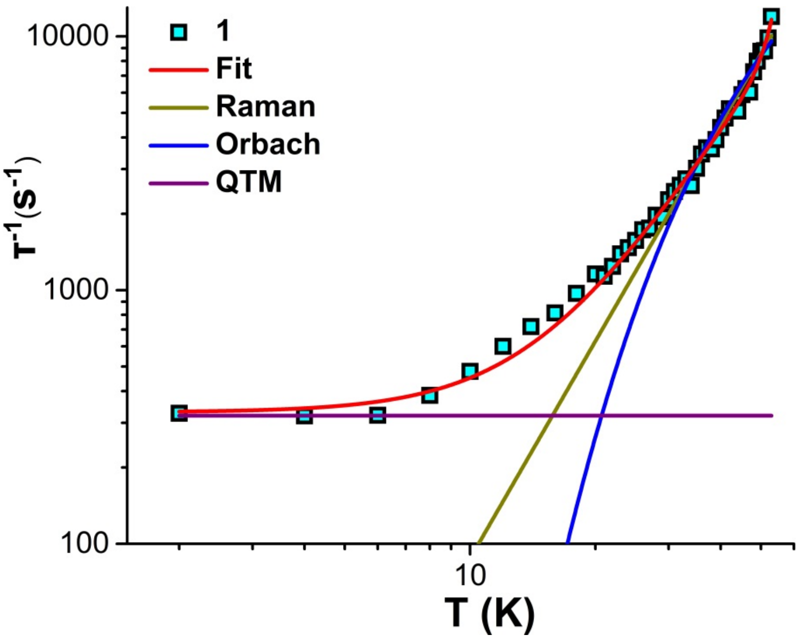

Figure S30. Log-Log plot of the relaxation times, τ^{-1} versus T for **1**. The data were analysed using the equation: $\tau^{-1} = \tau_{QTM}^{-1} + CT^n + \tau_0^{-1}\exp(-U_{eff}/T)$. The best fit (red line) gives n = 2.5, C = 0.37 K^{-n} s^{-1}, τ_{QTM} = 0.0030 s , τ_0 = 0.317 x 10^{-11} s and U_{eff} = 973 K.

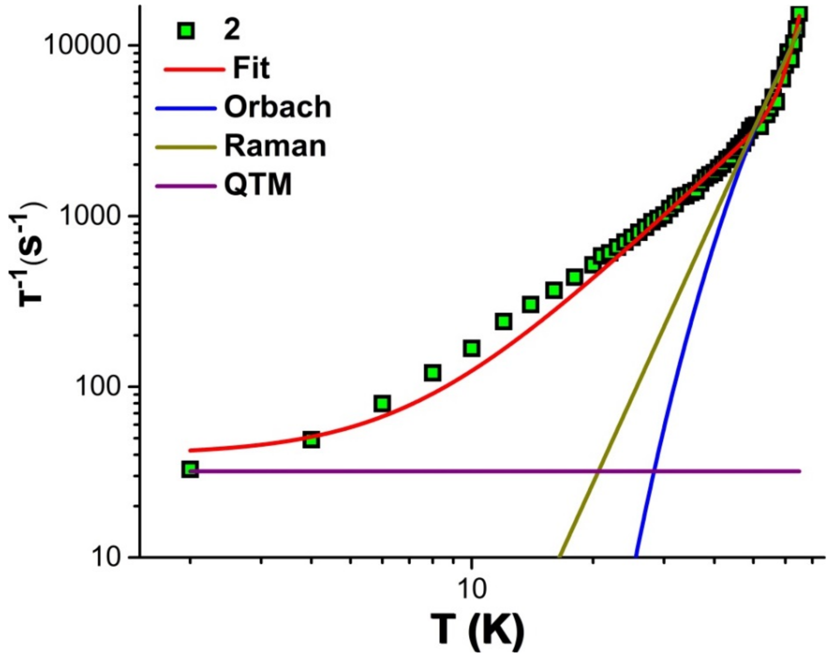

Figure S31. Log-Log plot of the relaxation times, τ^{-1} versus T for **2**. The data were analysed using the equation: $\tau^{-1} = \tau_{QTM}^{-1} + CT^n + \tau_0^{-1}\exp(-U_{eff}/T)$. The best fit (red line) gives n = 2.32, C = 0.34 K^{-n} s^{-1}, τ_{QTM} = 0.025 s , τ_0 = 0.196 x 10^{-10} s and U_{eff} = 1080 K.

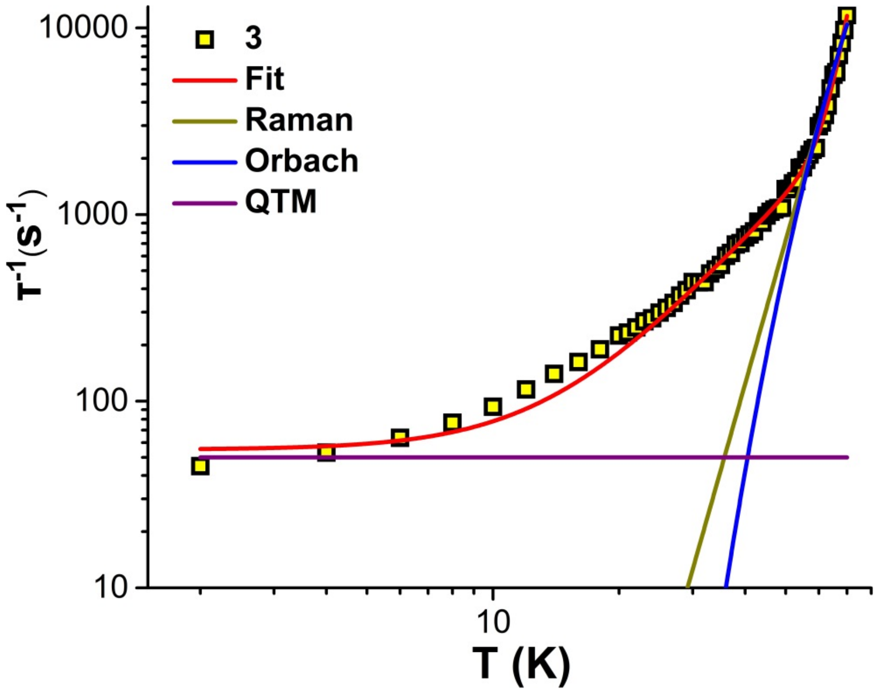

Figure S32. Log-Log plot of the relaxation times, τ^{-1} versus T for **3**. The data were analysed using the equation: $\tau^{-1} = \tau_{QTM}^{-1} + CT^{n} + \tau_0^{-1}\exp(-U_{eff}/T)$. The best fit (red line) gives n = 2.95, C = 0.014 $K^{-n}\,s^{-1}$, τ_{QTM} = 0.016 s , τ_0 = 0.152 x 10^{-10} s and U_{eff} = 1124K.

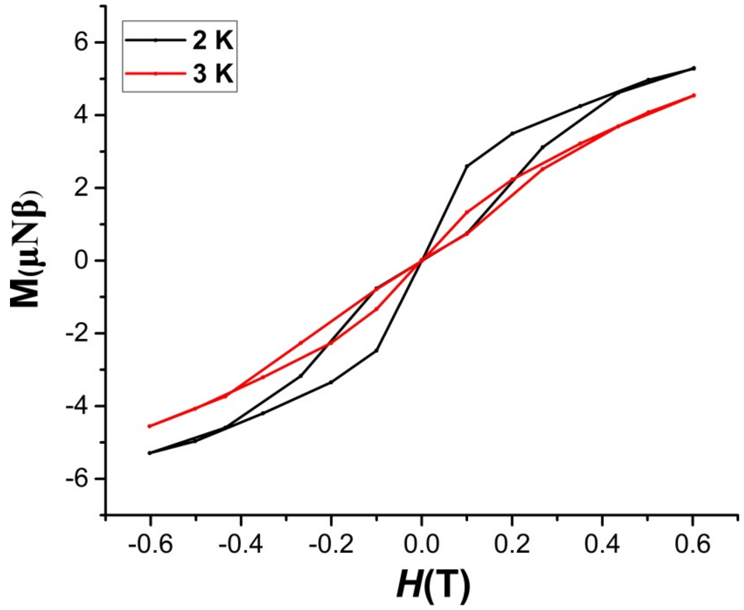

Figure S33. Powder magnetic hysteresis measurements for **1** with an average sweep rate of 4 mTs^{-1}.

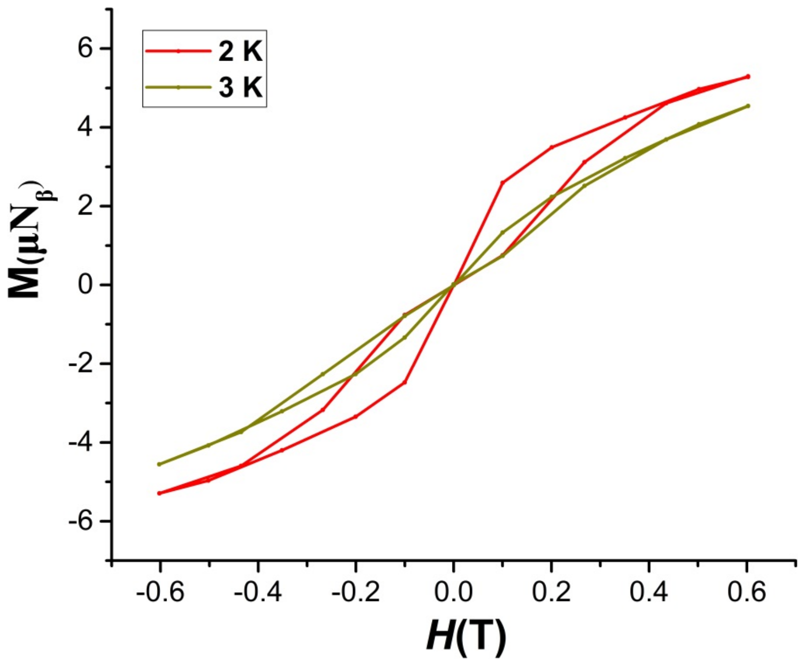

Figure S34. Powder magnetic hysteresis measurements for **2** with an average sweep rate of 4 mTs^{-1}.

4. Ab initio calculations

Computational Details:

To find the magnetic anisotropy of the metal centre in all the complexes *ab initio* CASSCF+SO-RASSI calculations have been performed using the MOLCAS 8.2 program package on the X-ray crystal structures of **1-3**. The relativistic effect of the Dy centre has been taken into account by the DKH Hamiltonian. Disk space for calculation of two-electron integrals has been reduced by the Cholesky decomposition technique.[4] The basis set for all the atoms has been taken from the ANO-RCC library implemented in the MOLCAS 8.2 program package. The basis set of VTZP quality was used for Dy, O, N atoms and VDZP was used for Si, P, F and C atoms. The basis set of VDZ quality was used for the hydrogen atoms. The active space for the Dy(III) ion contains nine electrons in seven orbitals; *i.e.* CAS (9.7). The sextet state of Dy(III) was optimized using 21 roots. The consideration of only the sextet state is enough to reproduce the experimental results as has been seen previously.[5] The twenty-one sextets have been mixed via SO-RASSI to calculate the spin-orbit coupling of the Dy(III) center. Finally, the g tensors and blocking barriers were calculated using the SINGLE_ANISO programme which uses the energy of the spin orbit states generated from RASSI-SO.[6] The corresponding bond lengths for the new *in silico* model **3c** are similar to the literature values.[7]

Table S8. CASSCF+RASSI-SO computed relative energies of the eight low lying Kramers Doublets along with g tensors and deviations from the principal magnetization axis with respect to the first KD for complex **1**.

Energy (K)	g_{xx}	g_{yy}	g_{zz}	Angle of g_{zz} between ground and higher excited state (°)
0	0.001	0.002	19.978	
620.1	0.217	0.242	16.988	4.670
1073.9	0.677	1.923	12.687	12.933
1271.5	1.557	3.539	10.737	83.509
1358.7	0.904	1.087	10.537	105.155
1404.6	0.122	5.589	13.765	97.685
1507.3	3.537	5.091	6.688	88.022
1631.2	1.638	3.890	15.277	72.831

Table S9. CASSCF+RASSI-SO computed relative energies of the eight low lying Kramers Doublets along with g tensors and deviations from the principal magnetization axis with respect to the first KD for complex **2**.

Energy (K)	g_{xx}	g_{yy}	g_{zz}	Angle of g_{zz} between ground and higher excited state (°)
0	0.001	0.002	19.992	
641.6	0.183	0.203	16.982	0.646
1137.8	0.614	1.695	12.766	2.760
1352.5	1.577	3.825	11.132	92.049
1434.8	0.041	3.722	10.174	86.840
1495.9	0.039	2.089	15.022	87.659
1594.3	1.214	3.414	8.712	90.237
1715.6	1.962	4.764	14.161	93.343

Table S10. CASSCF+RASSI-SO computed relative energies of the eight low lying Kramers Doublets along with g tensors and deviations from the principal magnetization axis with respect to the first KD for complex **3**.

Energy (K)	g_{xx}	g_{yy}	g_{zz}	Angle of g_{zz} between ground and higher excited state (°)
0	0.001	0.001	19.979	
671.9	0.144	0.152	16.987	0.968
1208.2	0.117	0.731	13.342	2.407
1484.2	1.608	3.538	6.541	84.987
1555.8	9.453	5.463	1.183	1.863
1598.9	0.141	5.832	12.493	94.991
1689.7	4.513	3.286	1.573	66.833
1813.3	2.252	6.441	12.746	83.665

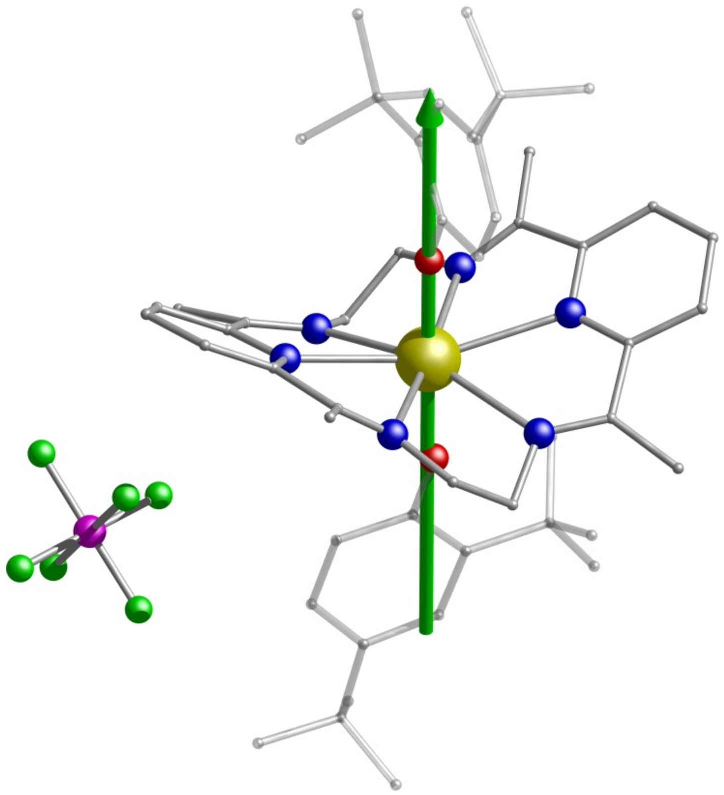

Figure S35. The direction of the principal anisotropy axis of the ground Kramers doublet for **1**. Colour code: Colour code: Dy, gold; O, red; N, blue, P, pink; C, grey; F, green. Hydrogens are omitted for clarity.

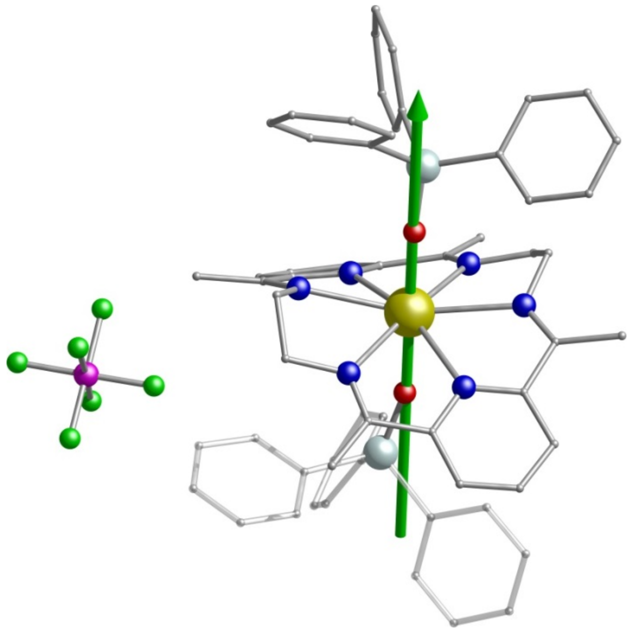

Figure S36. The direction of the principal anisotropy axis of the ground Kramers doublet for **2**. Colour code: Colour code: Dy, gold; O, red; N, blue, P, pink; C, grey; F, green. Hydrogens are omitted for clarity.

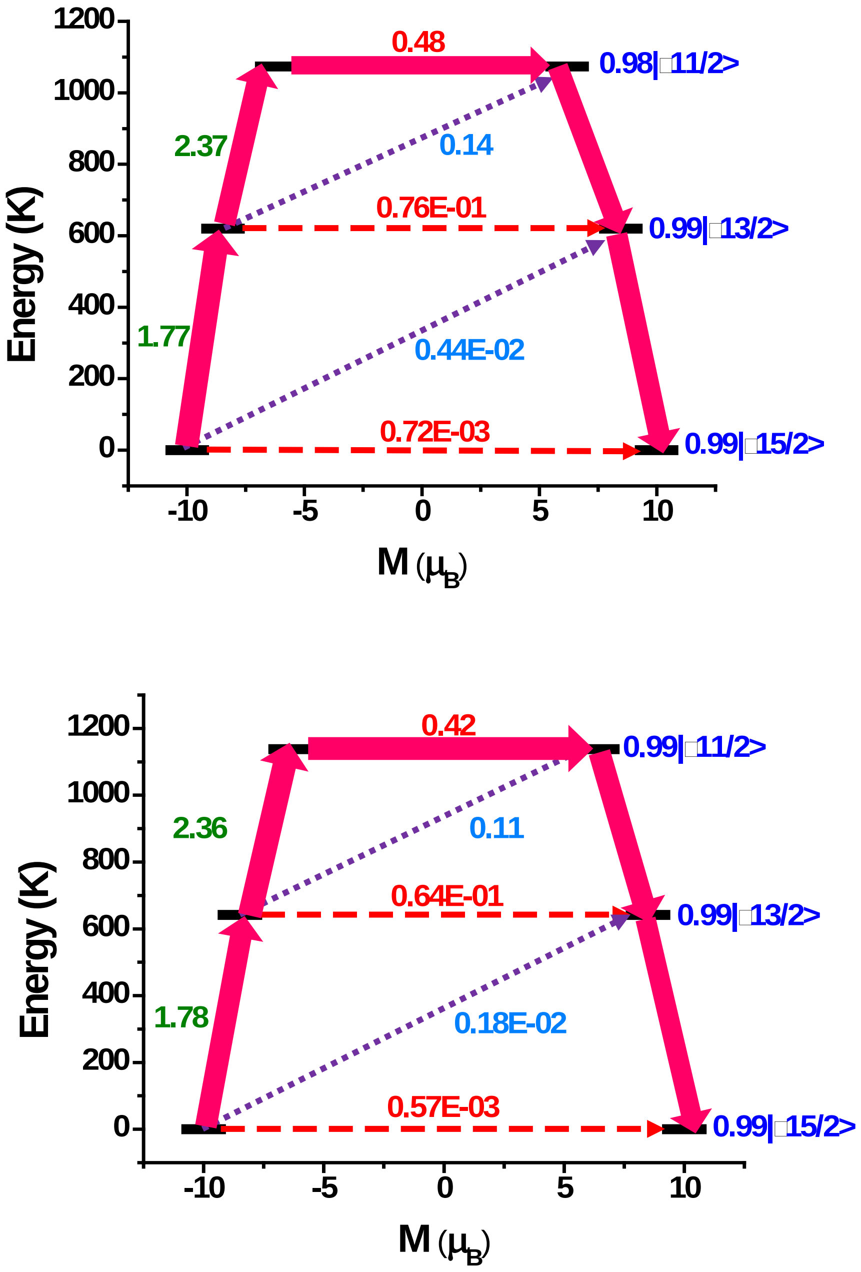

Figure S37. *Ab initio* calculated relaxation dynamics for complex **1** (upper) and **2** (lower). The arrows show the connected energy states with the number representing the matrix element of the transverse moment (see text for details). The black line indicates the KDs as function of magnetic moments. The red dashed arrow represents QTM (QTM = quantum tunnelling of the magnetisation) via the ground state and TA-QTM (TA-QTM = thermally assisted QTM) via excited states. The violet dotted arrow indicates possible Orbach process. The pink thick arrow indicates the mechanism of magnetic relaxation. The numbers above each arrow represent corresponding transverse matrix elements for the transition magnetic moments.

Table S11. LoProp charges of the atoms attached to the Dy centre of complexes **1**-**3**.[8]

Atoms	LoProp charge (1)	LoProp charge (2)	LoProp charge (3)
Dy	2.4543	2.4716	2.4684
O1	-0.9399	-1.2105	-1.2176
O2	-0.9613	-1.1977	-1.2177
N1	-0.3122	-0.3158	-0.3218
N2	-0.3639	-0.3536	-0.3764
N3	-0.3390	-0.3405	-0.3365
N4	-0.3064	-0.3088	-0.3066
N5	-0.3490	-0.3498	-0.3481
N6	-0.3777	-0.3566	-0.3663

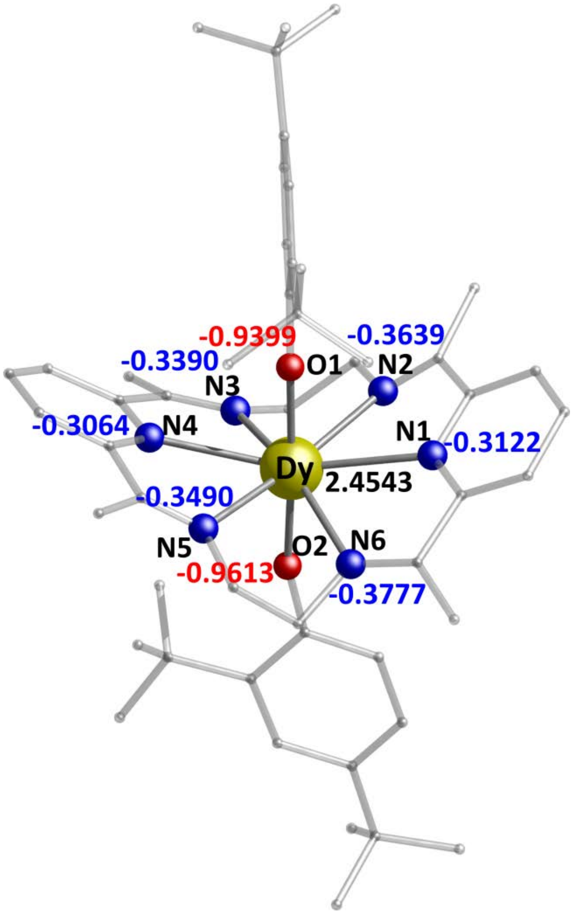

Figure S38. LoProp charges of the atoms attached to the Dy centre for complex **1**. Colour code: Dy, gold; O, red; N, blue, C, grey. Hydrogen atoms are omitted for clarity.

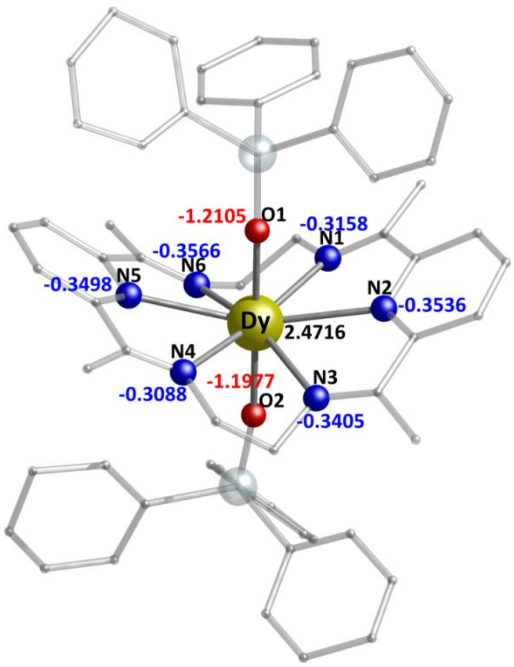

Figure S39. LoProp charges of the atoms attached to the Dy centre for complex **2**. Colour code: Dy, gold; O, red; N, blue, Si, light turquoise; C, grey. Hydrogen atoms are omitted for clarity.

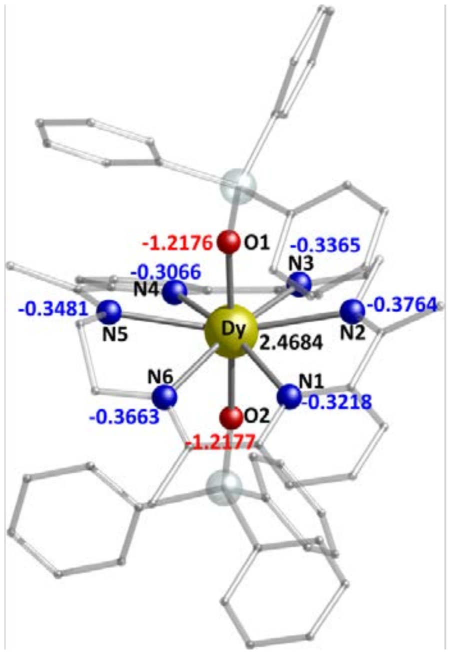

Figure S40. LoProp charges of the atoms attached to the Dy centre for complex **3**. Colour code: Dy, gold; O, red; N, blue, Si, light turquoise; C, grey. Hydrogen atoms are omitted for clarity.

Table S12. The *ab initio* computed crystal field parameters of complexes **1-3**.

k	q	B_k^q (1)	B_k^q (2)	B_k^q (3)
2	-2	6.90E-01	2.57E-02	3.01E-01
	-1	-1.63E+00	-6.31E-01	3.53E-01
	0	**-5.53E+00**	**-5.93E+00**	**-6.45E+00**
	1	-1.16E+00	9.49E-02	3.5-01
	2	5.55E-01	9.70E-01	3.93E-01
4	-4	-2.67E-03	-9.75E-05	-2.37E-04
	-3	7.75E-03	7.20E-03	2.48E-03
	-2	3.12E-04	-1.18E-03	-9.98E-04
	-1	4.85E-03	6.26E-03	-6.41E-04
	0	**-1.35E-02**	**-1.40E-02**	**-1.43E-02**
	1	5.68E-03	-3.25E-03	-2.92E-03
	2	-9.84E-05	-2.13E-04	1.27E-03
	3	2.32E-03	-6.68E-03	5.17E-03
	4	-4.80E-03	-6.30E-03	-2.51E-03
6	-6	5.99E-04	-6.99E-04	6.81E-04
	-5	3.77E-04	3.37E-05	-9.50E-05
	-4	-1.33E-05	-8.54E-07	-6.52E-07
	-3	1.57E-05	3.41E-05	4.52E-06
	-2	6.39E-06	-1.49E-05	-5.41E-06
	-1	1.22E-04	-3.61E-05	-3.35E-05
	0	**3.70E-05**	**4.39E-05**	**4.74E-05**
	1	4.17E-05	4.86E-05	7.35E-06
	2	2.10E-05	8.38E-07	3.50E-06
	3	-5.24E-06	-2.53E-05	2.42E-05
	4	-3.23E-05	-4.61E-05	-2.65E-05
	5	7.65E-05	-7.30E-05	-4.50E-05
	6	-3.53E-04	-5.75E-05	4.19E-05

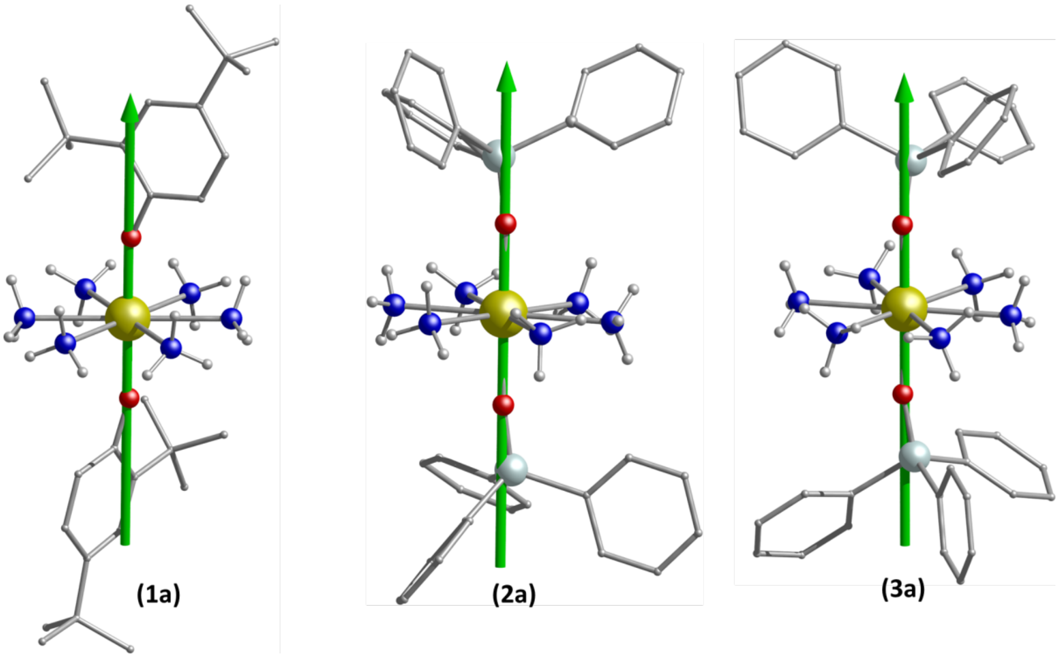

Figure S41. The magnetic anisotropy axis (green arrow) of the new *in-silico* models **1a**, **2a** and **3a**, where the L^{N6} ligand is replaced with six less bulky NH_3 groups. Colour code: Dy, gold; Si, light blue; O, red; N, blue; C, grey. Hydrogens are omitted for clarity.

Table S13. CASSCF+RASSI-SO computed relative energies of the eight low lying Kramers Doublets along with g tensors and deviations from the principal magnetization axis with respect to the first KD for model complex **1a**.

Energy (K)	g_x	g_y	g_z	Angle of g_{zz} between ground and higher excited state(°)
0	0.002	0.002	19.971	
606.3	0.532	0.576	16.787	6.490
957.6	0.085	4.574	9.418	34.407
1066.5	5.952	4.677	0.437	20.377
1132.4	0.486	2.279	12.628	102.962
1174.9	8.461	6.692	2.804	14.001
1331.4	6.528	6.196	7.712	39.089
1434.6	1.649	3.369	15.392	65.225

Table S14. CASSCF+RASSI-SO computed relative energies of the eight low lying Kramers Doublets along with g tensors and deviations from the principal magnetization axis with respect to the first KD for model complex **2a**.

Energy (K)	g_x	g_y	g_z	Angle of g_{zz} between ground and higher excited state(°)
0	0.001	0.002	19.981	
628.9	0.437	0.499	16.806	0.829
1023.5	7.784	6.218	0.149	84.657
1135.4	5.767	4.068	1.013	25.177
1215.9	1.839	2.199	7.349	87.830
1264.1	0.008	0.385	15.909	89.467
1405.3	4.553	5.989	8.113	92.106
1504.4	1.986	2.488	15.178	90.731

Table S15. CASSCF+RASSI-SO computed relative energies of the eight low lying Kramers Doublets along with g tensors and deviations from the principal magnetization axis with respect to the first KD for model complex **3a**.

Energy (K)	g_x	g_y	g_z	Angle of g_{zz} between ground and higher excited state(°)
0	0.000	0.000	19.977	
693.8	0.290	0.301	16.904	2.258
1250.3	0.492	2.033	11.362	9.468
1548.5	0.677	2.093	5.565	88.480
1623.6	0.958	5.090	9.629	93.523
1697.8	11.871	6.786	0.433	7.792
1762.9	1.537	2.959	5.365	20.056
1886.5	11.756	7.304	2.833	18.766

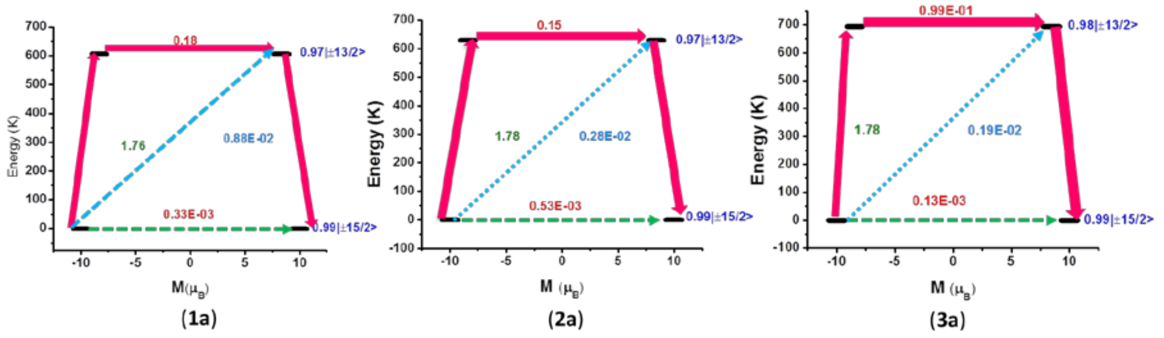

Figure S42. Ab initio calculated relaxation dynamics for *in-silico* models **1a**, **2a** and **3a**. The Black line indicates the KDs as function of magnetic moments. The green dashed arrows represent QTM via ground states. The sky blue dotted arrows indicate possible Orbach process. The pink thick arrows indicate the mechanism of magnetic relaxation. The numbers above each arrow represent corresponding transverse matrix elements for the transition magnetic moments.

Table S16. LoProp charges of the atoms attached to the Dy centre of model complexes **1a**-**3a**. [8]

Atoms	LoProp charge (1a)	LoProp charge (2a)	LoProp charge (3a)
Dy	2.4842	2.5032	2.5007
O1	-0.9612	-1.2260	-1.2327
O2	-0.9698	-1.2090	-1.2300
N1	-0.6263	-0.6391	-0.6401
N2	-0.6287	-0.6412	-0.6372
N3	-0.6332	-0.6347	-0.6356
N4	-0.6350	-0.6312	-0.6272
N5	-0.6386	-0.6373	-0.6329
N6	0.6357	-0.6382	-0.6296

Table S17. The *ab initio* computed crystal field parameters of model complexes **1a**-**3a**.

k	q	B_k^q (1a)	B_k^q (2a)	B_k^q (3a)
2	-2	-4.36E-02	6.19E-03	3.26E-01
	-1	7.52E-01	-1.98E-01	1.04E+00
	0	**-4.45E+00**	**-4.82E+00**	**-5.50E+00**
	1	2.20E+00	-5.02E-02	-3.40E-01
	2	4.79E-01	9.14E-01	-5.15E-02
4	-4	1.95E-03	-9.39E-04	-1.02E-03
	-3	2.22E-04	5.71E-03	8.51E-03
	-2	6.91E-04	2.40E-04	-9.70E-04
	-1	6.92E-04	2.66E-03	-4.62E-03
	0	**-1.50E-02**	**-1.55E-02**	**-1.57E-02**
	1	-9.07E-03	-2.06E-03	8.54E-04
	2	-2.28E-03	-2.43E-03	-1.72E-05
	3	-7.26E-03	-4.74E-03	-8.96E-04
	4	-1.34E-03	-2.95E-03	4.60E-04
6	-6	-1.76E-04	-7.79E-04	-7.36E-04
	-5	1.73E-04	-2.13E-05	-3.19E-05
	-4	8.68E-06	-7.78E-06	-1.37E-05
	-3	-1.12E-06	3.01E-05	9.16E-06
	-2	-2.08E-05	-6.48E-07	-1.06E-05
	-1	-1.14E-04	-2.62E-05	-4.68E-05
	0	**2.72E-05**	**3.49E-05**	**3.97E-05**
	1	-1.11E-04	4.87E-05	2.55E-05
	2	-6.98E-06	-2.29E-05	-8.66E-06
	3	-3.44E-05	-2.05E-05	6.89E-06
	4	-9.94E-06	-2.52E-05	7.52E-06
	5	-3.43E-04	-2.01E-05	1.65E-04
	6	7.76E-04	-1.69E-04	-1.98E-04

Table S18. CASSCF+RASSI-SO computed relative energies of the eight low lying Kramers Doublets along with g tensors and deviations from the principal magnetization axis with respect to the first KD for model complex **1b**.

Energy (K)	g_x	g_y	g_z	Angle of g_{zz} between ground and higher excited state (°)
0	0.001	0.002	19.979	
625.7	0.211	0.231	16.990	4.508
1084.1	0.573	1.692	12.798	12.103
1289.8	1.929	3.214	9.673	83.225
1368.9	0.506	1.071	10.150	103.544
1412.1	0.159	5.426	13.786	96.982
1517.7	5.781	4.663	3.300	25.114
1632.9	1.862	4.847	14.408	73.052

Table S19. CASSCF+RASSI-SO computed relative energies of the eight low lying Kramers Doublets along with g tensors and deviations from the principal magnetization axis with respect to the first KD for model complex **2b**.

Energy (K)	g_x	g_y	g_z	Angle of g_{zz} between ground and higher excited state (°)
0	0.001	0.002	19.985	
648.9	0.176	0.194	16.978	0.619
1153.9	0.576	1.545	12.901	2.556
1381.3	1.859	3.516	11.006	92.058
1467.2	0.566	3.552	9.694	85.432
1518.7	0.190	3.671	14.020	87.563
1616.9	1.320	3.326	8.240	90.300
1739.6	1.988	4.908	14.083	93.909

Table S20. CASSCF+RASSI-SO computed relative energies of the eight low lying Kramers Doublets along with g tensors and deviations from the principal magnetization axis with respect to the first KD for model complex **3b**.

Energy (K)	g_x	g_y	g_z	Angle of g_{zz} between ground and higher excited state (°)
0	0.001	0.001	19.983	
682.1	0.135	0.141	16.991	0.888
1229.9	0.094	0.615	13.445	2.113
1521.2	5.166	3.972	1.348	92.066
1595.3	9.342	5.897	0.996	2.709
1638.3	0.084	4.585	12.757	93.607
1726.7	1.326	2.777	4.242	96.358
1850.1	2.172	6.856	12.393	84.724

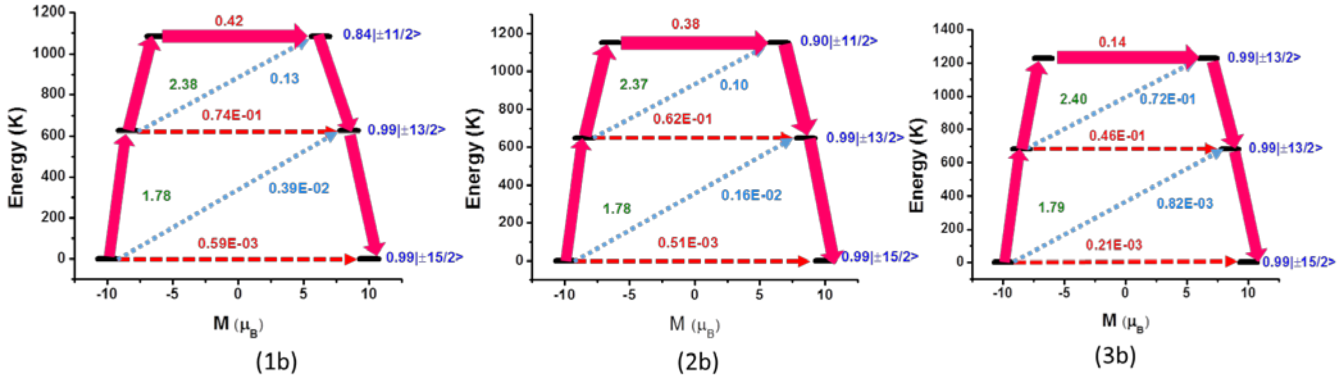

Figure S43. Ab initio calculated relaxation dynamics for *in-silico* models **1b**, **2b** and **3b**. The Black line indicates the KDs as function of magnetic moments. The red dashed arrow represents QTM (QTM = quantum tunnelling of the magnetisation) via the ground state and TA-QTM (TA-QTM = thermally assisted QTM) via excited states. The sky blue dotted arrows indicate possible Orbach process. The pink thick arrows indicate the mechanism of magnetic relaxation. The numbers above each arrow represent corresponding transverse matrix elements for the transition magnetic moments.

Table S21. LoProp charges of the atoms attached to the Dy centre of model complexes **1b**-**3b**. [8]

Atoms	LoProp charge (1b)	LoProp charge (2b)	LoProp charge (3b)
Dy	2.4539	2.4721	2.4688
O1	-0.9474	-1.2151	-1.2207
O2	-0.9562	-1.1963	-1.2200
N1	-0.3129	-0.3148	-0.3171
N2	-0.3576	-0.3454	-0.3688
N3	-0.3524	-0.3468	-0.3426
N4	-0.3114	-0.3084	-0.3082
N5	-0.3650	-0.3523	-0.3563
N6	-0.3552	-0.3574	-0.3530

Table S22. The *ab initio* computed crystal field parameters of model complexes **1b**-**3b**.

k	q	B_k^q (1b)	B_k^q (2b)	B_k^q (3b)
2	-2	5.43E-01	-2.12E-01	3.77E-01
	-1	1.48E+00	-5.82E-01	-2.92E-01
	0	**-5.58E+00**	**-6.05E+00**	**-6.64E+00**
	1	1.15E+00	5.59E-02	-3.11E-01
	2	4.64E-01	8.73E-01	2.40E-01
4	-4	-2.49E-03	1.34E-03	-7.73E-04
	-3	-7.70E-03	8.05E-03	-3.27E-03
	-2	3.40E-04	-1.14E-03	-9.19E-04
	-1	-3.72E-03	5.68E-03	7.14E-05
	0	**-1.36E-02**	**-1.41E-02**	**-1.43E-02**
	1	-5.32E-03	-2.72E-03	2.66E-03
	2	3.63E-05	-3.39E-04	1.42E-03
	3	-2.26E-03	-5.69E-03	-5.01E-03
	4	-4.89E-03	-5.98E-03	-2.21E-03
6	-6	6.34E-04	-6.39E-04	6.67E-04
	-5	-3.69E-04	5.66E-05	1.03E-04
	-4	-1.17E-05	9.26E-06	-6.73E-06
	-3	-2.00E-05	3.85E-05	-5.58E-06
	-2	3.37E-06	-1.32E-05	-5.23E-06
	-1	-1.24E-04	-3.53E-05	3.62E-05
	0	**3.66E-05**	**4.44E-05**	**4.80E-05**
	1	-4.59E-05	4.55E-05	-7.78E-06
	2	2.43E-05	-8.22E-07	4.47E-06
	3	5.26E-06	-2.25E-05	-1.90E-05
	4	-3.00E-05	-4.50E-05	-2.61E-05
	5	-1.01E-04	-7.52E-05	3.67E-05
	6	-2.80E-04	-2.89E-04	-1.50E-04

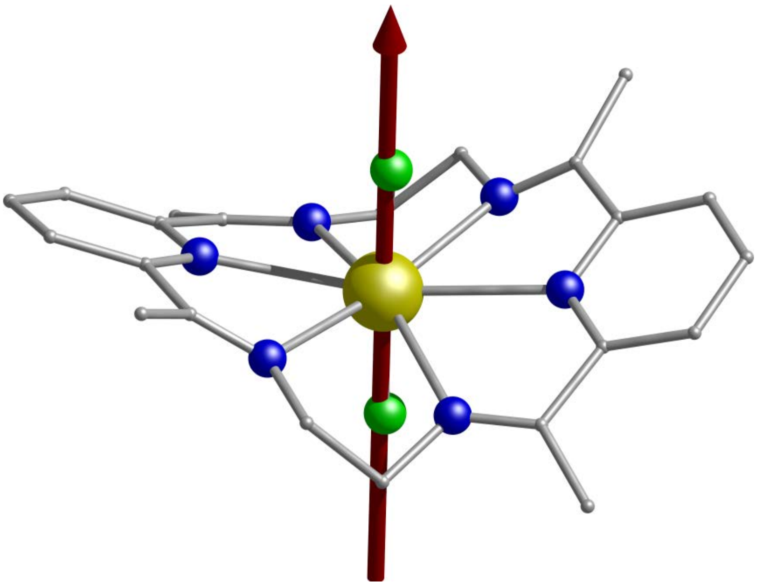

Figure S44. The magnetic anisotropy axis (brown arrow) of the new *in silico* model **3c**, derived from complex **3** where the axial ligands have been replaced *in silico* by the strong electronegative F^{-} ions. Colour code: Dy, gold; F, green; O, red; N, blue; C, grey. Hydrogens are omitted for clarity.

Table S23. CASSCF+RASSI-SO computed relative energies of the eight low lying Kramers Doublets along with g tensors and deviations from the principal magnetization axis with respect to the first KD for model complex **3c**.

**Energy (K)**	g_x	g_y	g_z	**Angle of g_{zz} between ground and higher excited state(°)**
0	0.001	0.001	19.983	
648.9	0.148	0.154	16.985	0.496
1193.6	0.019	0.891	13.075	1.850
1466.5	4.932	3.503	1.081	12.586
1502.9	0.964	3.995	11.071	90.919
1585.2	11.825	6.975	0.628	5.456
1670.8	2.476	3.643	4.892	19.215
1808.4	2.915	6.010	12.715	80.501

Table S24. LoProp charges of the atoms attached to the Dy centre of model complex **3c**. [8]

**Atom**	**3c**
Dy	2.5015
F^{-}	-0.9164
F^{-}	-0.9191
N2	-0.3184
N3	-0.3653
N4	-0.3508
N5	-0.3143
N6	-0.3644
N7	-0.3512

Table S25. The *ab initio* computed crystal field parameters of model complexes **3c**.

k	q	B_k^q (**3c**)
2	-2	4.40E-01
	-1	2.85E-01
	**0**	**-6.49E+00**
	1	3.91E-01
	2	1.15E-01
4	-4	-3.75E-04
	-3	5.96E-03
	-2	-7.09E-04
	-1	-2.19E-03
	**0**	**-1.54E-02**
	1	-2.86E-03
	2	3.23E-03
	3	1.22E-03
	4	-1.32E-04
6	-6	6.53E-04
	-5	-1.27E-04
	-4	-1.76E-05
	-3	-2.29E-05
	-2	-1.54E-05
	-1	8.38E-06
	**0**	**6.04E-05**
	1	1.51E-06
	2	3.85E-06
	3	1.66E-05
	4	-7.23E-05
	5	6.67E-05
	6	-2.12E-04

References

[1] L. De Cola, D. L. Smailes, L. M. Vallarino, *Inorg.Chem.* **1986**, *25*, 1729-1732.

[2] S. P. Westrip, *J. Apply. Cryst.* **2010**, *43*, 920.

[3] a) M. Llunell, D. Casanova, J. Cirera, P. Alemany and S. Alvarez, Shape Program, Version 2.0, 2010; b) D. Casanova, M. Llunell, P. Alemany, S. Alvarez. *Chem. Eur. J.* **2005**, *11*, 1479.

[4] a) F. Aquilante, J. Autschbach, K. Carlson Rebecca, F. Chibotaru Liviu, G. Delcey Mickaël, L. De Vico, I. Fdez. Galván, N. Ferré, M. Frutos Luis, L. Gagliardi, M. Garavelli, A. Giussani, E. Hoyer Chad, G. Li Manni, H. Lischka, D. Ma, Å. Malmqvist Per, T. Müller, A. Nenov, M. Olivucci, B. Pedersen Thomas, D. Peng, F. Plasser, B. Pritchard, M. Reiher, I. Rivalta, I. Schapiro, J. Segarra-Martí, M. Stenrup, G. Truhlar Donald, L. Ungur, A. Valentini, S. Vancoillie, V. Veryazov, P. Vysotskiy Victor, O. Weingart, F. Zapata, R. Lindh, *J. Comput. Chem*., **2015**, *37*, 506-541; b) F. Aquilante, L. De Vico, N. Ferré, G. Ghigo, P.-a. Malmqvist, P. Neogrády, T. B. Pedersen, M. Pitoňák, M. Reiher, B. O. Roos, L. Serrano-Andrés, M. Urban, V. Veryazov, R. Lindh, *J. Comput. Chem*., **2010**, 31, 224-247; c) J. A. Duncan, *J. Am. Chem. Soc*., **2009**, 131, 2416-2416; d) V. Veryazov, P. O. Widmark, L. Serrano-Andrés, R. Lindh, O. Roos Björn, *Int. J. Quantum Chem*., **2004**, *100*, 626-635; e) G. Karlström, R. Lindh, P.-Å. Malmqvist, B. O. Roos, U. Ryde, V. Veryazov, P.-O. Widmark, M. Cossi, B. Schimmelpfennig, P. Neogrady and L. Seijo, *Comput. Mater. Sci*., **2003**, 28, 222-239; f) L. F. Chibotaru and L. Ungur, *J. Chem. Phys*., **2012**, *137*, 064112; g) F. Habib, R. Luca Oana, V. Vieru, M. Shiddiq, I. Korobkov, I. Gorelsky Serge, K. Takase Michael, F. Chibotaru Liviu, S. Hill, H. Crabtree Robert, M. Murugesu, *Angew. Chem. Int. Ed*., **2013**, *52*, 11290-11293; h) S. K. Langley, L. Ungur, N. F. Chilton, B. Moubaraki, L. F. Chibotaru, K. S. Murray, *Inorg. Chem.,* **2014**, *53*, 4303-4315. I) B. O. Roos, R. Lindh, P.-Å. Malmqvist, V. Veryazov, P.-O. Widmark and A. C. Borin, *J. Phys. Chem. A*, **2008,** *112*, 11431-11435.

[5] a) K. Bernot, J. Luzon, L. Bogani, M. Etienne, C. Sangregorio, M. Shanmugam, A. Caneschi, R. Sessoli, D. Gatteschi, *J. Am. Chem. Soc.* **2009**, *131*, 5573–5579; b) A. Upadhyay, S. K. Singh, C. Das, R. Mondol, S. K. Langley, K. S. Murray, G. Rajaraman, M. Shanmugam, *Chem. Commun.* **2014**,50, 8838-8841.

[6] L. Ungur, M. Thewissen, J. P. Costes, W. Wernsdorfer, L. F. Chibotaru, *Inorg. Chem*., **2013**, 52, 6328.

[7] A. B. Canaj, M. K. Singh, E. R. Marti, M. Damjanović, C. Wilson, O. Céspedes, W. Wernsdorfer, G. Rajaraman, M. Murrie, *Chem. Commun*. **2019**, *55*, 5950.

[8] L. Gagliardi, R. Lindh and G. Karlstrom, *J. Chem. Phys*., **2004**, *121*, 4494-4500.
